# Efficacy of Losartan in Hospitalized Patients With COVID-19–Induced Lung Injury: A Randomized Clinical Trial

**DOI:** 10.1001/jamanetworkopen.2022.2735

**Published:** 2022-03-16

**Authors:** Michael A. Puskarich, Nicholas E. Ingraham, Lisa H. Merck, Brian E. Driver, David A. Wacker, Lauren Page Black, Alan E. Jones, Courtney V. Fletcher, Andrew M. South, Thomas A. Murray, Christopher Lewandowski, Joseph Farhat, Justin L. Benoit, Michelle H. Biros, Kartik Cherabuddi, Jeffrey G. Chipman, Timothy W. Schacker, Faheem W. Guirgis, Helen T. Voelker, Joseph S. Koopmeiners, Christopher J. Tignanelli

**Affiliations:** 1 Department of Emergency Medicine, University of Minnesota, Minneapolis; 2 Department of Emergency Medicine, Hennepin County Medical Center, Minneapolis, Minnesota; 3 Division of Pulmonary, Allergy, Critical Care and Sleep Medicine, Department of Medicine, University of Minnesota, Minneapolis; 4 Department of Emergency Medicine, University of Florida College of Medicine, Gainesville; 5 Department of Emergency Medicine, University of Florida College of Medicine, Jacksonville; 6 Department of Emergency Medicine, University of Mississippi Medical Center, Jackson; 7 Center for Drug Discovery, University of Nebraska Medical Center, Omaha; 8 Section of Nephrology, Department of Pediatrics, Wake Forest School of Medicine and Brenner Children's Hospital, Winston Salem, North Carolina; 9 Division of Public Health Sciences, Department of Epidemiology and Prevention, Wake Forest School of Medicine, Winston Salem, North Carolina; 10 Department of Surgery-Hypertension and Vascular Research, Wake Forest School of Medicine, Winston Salem, North Carolina; 11 Department of Biostatistics, School of Public Health, University of Minnesota, Minneapolis; 12 Department of Emergency Medicine, Henry Ford Hospital, Wayne State University, Detroit, Michigan; 13 Department of Surgery, North Memorial Medical Center, Minneapolis, Minnesota; 14 Department of Emergency Medicine, University of Cincinnati, Cincinnati, Ohio; 15 Department of Surgery, University of Minnesota, Minneapolis; 16 Division of Infectious Disease, Department of Medicine, University of Minnesota, Minneapolis

## Abstract

**Importance:**

SARS-CoV-2 viral entry may disrupt angiotensin II (AII) homeostasis, contributing to COVID-19 induced lung injury. AII type 1 receptor blockade mitigates lung injury in preclinical models, although data in humans with COVID-19 remain mixed.

**Objective:**

To test the efficacy of losartan to reduce lung injury in hospitalized patients with COVID-19.

**Design, Setting, and Participants:**

This blinded, placebo-controlled randomized clinical trial was conducted in 13 hospitals in the United States from April 2020 to February 2021. Hospitalized patients with COVID-19 and a respiratory sequential organ failure assessment score of at least 1 and not already using a renin-angiotensin-aldosterone system (RAAS) inhibitor were eligible for participation. Data were analyzed from April 19 to August 24, 2021.

**Interventions:**

Losartan 50 mg orally twice daily vs equivalent placebo for 10 days or until hospital discharge.

**Main Outcomes and Measures:**

The primary outcome was the imputed arterial partial pressure of oxygen to fraction of inspired oxygen (Pao_2_:Fio_2_) ratio at 7 days. Secondary outcomes included ordinal COVID-19 severity; days without supplemental o_2_, ventilation, or vasopressors; and mortality. Losartan pharmacokinetics and RAAS components (AII, angiotensin-[1-7] and angiotensin-converting enzymes 1 and 2)] were measured in a subgroup of participants.

**Results:**

A total of 205 participants (mean [SD] age, 55.2 [15.7] years; 123 [60.0%] men) were randomized, with 101 participants assigned to losartan and 104 participants assigned to placebo. Compared with placebo, losartan did not significantly affect Pao_2_:Fio_2_ ratio at 7 days (difference, −24.8 [95%, −55.6 to 6.1]; *P* = .12). Compared with placebo, losartan did not improve any secondary clinical outcomes and led to fewer vasopressor-free days than placebo (median [IQR], 9.4 [9.1-9.8] vasopressor-free days vs 8.7 [8.2-9.3] vasopressor-free days).

**Conclusions and Relevance:**

This randomized clinical trial found that initiation of orally administered losartan to hospitalized patients with COVID-19 and acute lung injury did not improve Pao_2_:Fio_2_ ratio at 7 days. These data may have implications for ongoing clinical trials.

**Trial Registration:**

ClinicalTrials.gov Identifier: NCT04312009

## Key Points

**Question:**

What is the effect of losartan on lung injury in hospitalized patients with COVID-19?

**Findings:**

In this randomized clinical trial in 205 patients with evidence of COVID-19–induced acute lung injury, angiotensin receptor blockade with maximal dose losartan did not reduce lung injury at 7 days, as measured by partial pressure of oxygen to fraction of inspired oxygen ratio. Secondary outcomes, including ventilator-free days and mortality, were unaffected, but patients treated with losartan had fewer vasopressor-free days.

**Meaning:**

This randomized clinical trial found that losartan for angiotensin receptor blockade did not reduce lung injury in patients with COVID-19 and raised concerns about risks of harm.

## Teaser

This randomized clinical trial tests the efficacy of losartan to reduce lung injury in hospitalized patients with COVID-19.

## Introduction

SARS-CoV-2 has infected more than 195 million persons, causing more than 4 million deaths.^1^ SARS-CoV-2 enters respiratory epithelial cells via angiotensin-converting enzyme 2 (ACE2), a major component of the renin-angiotensin-aldosterone system (RAAS).^2,3^ ACE2 is responsible for degradation of angiotensin II (AII), a proinflammatory vasoconstrictor, into angiotensin-(1-7) and angiotensin-(1-9), which generally oppose AII’s effects.^2,4^ Early data suggested AII levels may be associated with viral load and degree of lung injury in patients with COVID-19,^4^ although more recent and methodologically robust data demonstrate mixed findings.^5,6,7^

Preclinical models of viral pneumonias affecting ACE2, including SARS-CoV-2, have demonstrated that AII type 1 receptor (AT1R) blockade reduces lung injury and death.^8,9,10^ Meanwhile, observational studies of the associations of antecedent angiotensin-converting enzyme inhibitor (ACE1) or AT1R blocker (ARB) use with disease severity and mortality in COVID-19 have reported mixed results.^11,12,13,14,15,16,17^ Some of these studies were limited by insufficient adjustment for confounders, confounding by indication, and lack of data regarding adherence, and most importantly, none assessed the effect of ACE or ARB initiation in otherwise naive patient populations. Therefore, RAAS inhibition in COVID-19 remains an ongoing area of controversy.

We hypothesized losartan treatment might reduce lung injury and improve clinical outcomes in hospitalized patients with COVID-19 by restoring AII and angiotensin-(1-7) homeostasis. To test this hypothesis, we conducted a multicenter, blinded, and placebo-controlled randomized clinical trial of patients hospitalized with COVID-19 not already using an RAAS inhibitor. The primary objective was to test if losartan improves the ratio of arterial partial pressure of oxygen to fraction of inspired oxygen (Pao_2_:Fio_2_) at 7 days. We also sought to determine if losartan affects biochemical markers (including RAAS components), severity of illness, and mortality.

## Methods

### Study Design

We conducted a prospective multicenter, blinded, and placebo-controlled randomized clinical trial of hospitalized patients with COVID-19 at 13 hospitals in the United States from April 2020 to February 2021. The trial was approved by a central institutional review board (Advarra) and conducted following good clinical practice guidelines under the oversight of an independent data safety monitoring board. The trial protocol and statistical analysis plan are provided in Supplement 1. All participants or their legally authorized representatives provided written electronic informed consent. The trial was conducted under the authority of the Food and Drug Administration and registered on ClinicalTrials.gov prior to initialization. This study is reported following the Consolidated Standards of Reporting Trials (CONSORT) reporting guideline.

### Participants

Consecutive patients presenting to a participating institution with a reverse transcriptase–polymerase chain reaction (RT-PCR) test result positive for SARS-CoV-2 were assessed for eligibility. Participants were eligible if they had at least 1 Centers for Disease Control and Prevention–recognized symptom of COVID-19, a positive clinical RT-PCR SARS-CoV-2 result, and a respiratory sequential organ failure assessment (SOFA) score of 1 or higher.^18^ Exclusion criteria included active outpatient treatment with an ACEI, ARB, or aliskiren; prior adverse reaction to ACEIs or ARBs; pregnancy, breastfeeding, or lack of contraception; history of dialysis, stage IV chronic kidney disease, or estimated glomerular filtration rate (eGFR) less than 30 mL/min/1.73 m^2^; potassium level greater than 5.0 mEq/L (to convert to millimoles per liter, multiply by 1); history of severe liver disease; enrollment in another blinded clinical trial for COVID-19; lack of informed consent; inability to randomize within 48 hours of admission or positive test result; no decrease in o_2_ saturation from baseline; or respiratory SOFA score of less than 1 (defined as Pao_2_:Fio_2_ <400, using arterial oxygen saturation if Pao_2_ data were unavailable).^19,20^ Race and ethnicity were self-identified by the patient at the time of hospital admission. Race and ethnicity were analyzed based on prior literature demonstrating differential outcomes associated with these factors.

### Screening and Consent

Electronic health records (EHRs) were manually screened by trained research personnel. Owing to limitations in personal protective equipment, informed consent was typically conducted remotely via telephone or video teleconference, and documentation was maintained using a 21 CFR part 11–adherent electronic consent platform (REDCap).^21^

### Randomization and Blinding

Enrolled participants were randomized using permuted blocks of randomly varying sizes (2, 4, or 6) stratified by site and age (≥60 or <60 years). Stratification by age was performed owing to markedly different COVID-19 outcomes by age, while site blocking was used to account for large regional differences in local practice patterns and potential resource capacity issues over the course of the pandemic. A central randomization website generated treatment allocations in a 1-to-1 ratio. All participants and study personnel were blinded except statisticians preparing the randomization and investigational pharmacists.

### Intervention

The intervention was losartan 50 mg orally twice daily (100 mg daily total) vs a visually indistinguishable placebo, yielding an expected 70% inhibition of AT1R.^22^ The study drug was shipped to sites as pills and prepared locally by an unblinded pharmacist in suspension, per the manufacturer’s package insert. The study drug was administered for 10 days for patients with eGFR greater than 60 mL/min/1.73 m^2^, once daily for those with eGFR 30 to 60 mL/min/1.73 m^2^, and discontinued if eGFR decreased below 30 mL/min/1.73 m^2^, following discharge, or by a blinded investigator if a drug-related serious adverse effect (SAE) was suspected.

### Primary Efficacy Outcome

The primary outcome was the worst recorded Pao_2_:FiO_2_ ratio on day 7. If lacking, Pao_2_ was calculated using the method of Pandharipande (for positive pressure ventilation) or Gadrey (no positive pressure).^20,23^ Participants discharged prior to day 7 were provided a home pulse oximeter and contacted via phone. This outcome was chosen early in the pandemic as a surrogate outcome related to respiratory failure, given early concerns surrounding capacity and ventilator shortages in resource-limited settings, including ventilatory shortages.

### Secondary Outcomes

Oxygen-, ventilator-, and vasopressor-free days (to 10 days); time to discharge; 7-point ordinal scale of COVID-19 severity^24^; and 28-day mortality were measured. A subset of participants (limited by local biohazard capacity) underwent biospecimen collection.

### Safety Monitoring

Creatinine and potassium were measured on days 0, 1, 3, and 7. Acute kidney injury was defined as an increase in serum creatinine of 0.3 mg/dL (to convert to micromoles per liter, multiply by 88.4) or 1.5-fold baseline values.^25^ Blood pressures and EHRs were reviewed daily for up to 15 days after randomization. Specific hold parameters for mean arterial pressure less than 65 mm Hg, decrease in eGFR to less than 30 mL/min/1.73 m^2^, and potassium level greater than 5.5 mEq/L prompted holding the study drug until these variables normalized. The drug could also be held or discontinued per investigator discretion.

### Pharmacokinetic Measurements

Blood samples were obtained at 2, 4, and 6 hours after a dose of losartan or placebo (50 mg) in a subgroup blinded to treatment allocation. Plasma concentrations of losartan and its active carboxy metabolite, carboxylosartan, were quantified by a validated liquid chromatography–mass spectrometry (LC-MS) or MS assay with a lower limit of quantification of 3 ng/mL at the University of Nebraska Medical Center.

### RAAS Measurements

Blood was collected in an EDTA tube containing a cocktail of protease inhibitors validated with the intended assays and red-top tubes per best practices on study days 1, 2, 4, 6, 8, 10, and 15, or until hospital discharge.^26,27,28^ Extracted plasma and serum were stored at −80 °C and sent to the Clinical Laboratory Improvement Amendments–certified Biomarker Analytical Core at Atrium Health Wake Forest Baptist for analysis. The plasma was thawed on ice, extracted on Sep-Pak C18 columns (Waters Corp), and the eluted fractions were analyzed with radioimmunoassays.^27^ Plasma AII was measured using Immuno-Biological Lab kits (IBL America), while plasma angiotensin-(1-7) was measured using an antibody produced by the laboratory and validated with LC-MS or MS. Serum ACE and ACE2 activity were analyzed using established methods.^26,27^

### Viral Load

Consistent with our prior work, we report normalized count values for SARS-CoV-2 targets compared with human RNase P gene internal sample control, providing a relative value to compensate for sampling and extraction quality.^29^

### Power and Sample Size

Owing to a paucity of data at study design, we based expected variance of Pao_2_:Fio_2_ on prior studies of viral-induced acute lung injury, considering SDs between 50 and 125.^30,31,32,33^ At a sample size of 200 with a 1-to-1 allocation ratio, we calculated 90% power to detect a difference of 70 in the Pao_2_:Fio_2_ ratio assuming an SD of 150, and 80% to detect a 50-unit difference, assuming an SD of 125.

### Statistical Analysis

Baseline clinical characteristics are summarized using descriptive statistics. All analyses were conducted using intent-to-treat principles. We used linear regression to evaluate the main effect of losartan on Pao_2_:Fio_2_ ratio at day 7 adjusted for Pao_2_:Fio_2_ ratio at baseline. Participants who died before day 7 were assigned a Pao_2_:Fio_2_ ratio of zero. Missing values were multiply imputed using predictive mean matching with predictive models that included study arm, location of enrollment, baseline hypertension, assigned sex, age, body mass index (BMI; calculated as weight in kilograms divided by height in meters squared), baseline Pao_2_:Fio_2_ ratio, and day from randomization to discharge. SEs were estimated using a heteroscedasticity-consistent covariance matrix.

Longitudinal secondary end points were analyzed using generalized linear mixed models or generalized estimating equations, adjusting for corresponding baseline measurements. Mortality was summarized using Kaplan-Meier plots and compared using the log-rank test and unadjusted Cox models to estimate hazard ratios. Time to hospital discharge, with death in hospital or discharge to comfort care as competing risks, was evaluated using cumulative incidence plots and Fine-Gray test (ρ = 0), the competing risks’ analog to the log-rank test. Ordinal outcomes were analyzed using proportional odds regression.

Pharmacokinetic characteristics were determined using the trapezoidal rule and linear regression (Phoenix WinNonLin version 8.3; Certara). RAAS data were analyzed by jointly modeling RAAS components and time-to-discharge using the JM package in R statistical software (version 4.0.3 or newer; R Project for Statistical Computing) on the natural-log scale using linear mixed effects models with terms for treatment assignment, baseline values, and a linear term for day. Viral load was analyzed using linear mixed models with a visit-by-treatment group interaction and a global Wald test for overall treatment statistical significance.

Statistical analyses were performed using SAS (version 9.4 or newer; SAS Institute) or R (R Project for Statistical Computing). All statistical tests were 2-tailed with *P* < .05 considered statistically significant. Data were analyzed from April 19 to August 24, 2021.

## Results

**Figure 1.**
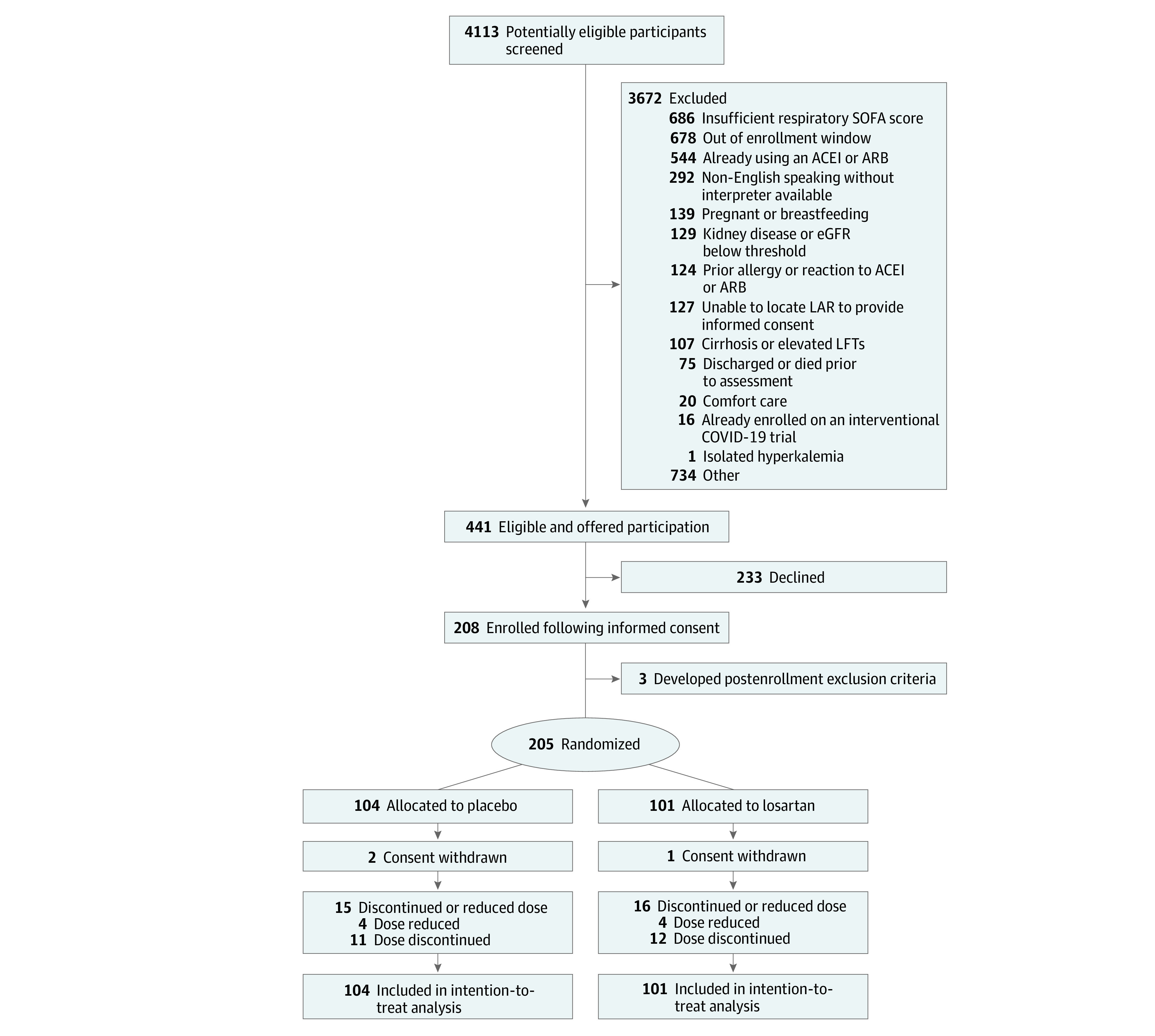
Participant Recruitment Flowchart ACEI indicates angiotensin-converting enzyme inhibitor; ARB, angiotensin II type 1 receptor blocker; eGFR, estimated glomerular filtration rate; LAR, legally authorized representative; LFT, liver function test; and SOFA, sequential organ failure assessment.

Of 4113 patients screened, 3672 met exclusion criteria (Figure 1). Of those eligible, 208 (47.1%) provided consent to participate. Three patients developed postconsent exclusions prior to randomization, leaving 205 randomized participants, with 104 assigned to placebo and 101 assigned to losartan, representing the intent-to-treat cohort. The overall mean (SD) age was 55.2 (15.7) years, and 123 participants [60.0%] were men. The cohort included diverse racial and ethnic representation (9 Asian patients [4.4%]; 67 Black patients [33.7%]; and 37 Hispanic patients18.0%]; 82 White patients [40.0%]), with a high prevalence of obesity (52 patients [25.4%] with BMI 25 to <30; 129 patients [62.9%] with BMI ≥30). All participants were followed until completion or withdrawal. Demographic and clinical characteristics were well matched (Table 1), although more patients in the losartan group than the placebo group were enrolled in the intensive care unit (ICU) (17 patients [16.8%] vs 9 patients [8.7%]).

**Table 1.** Participant Demographic and Clinical Characteristics

Characteristic	No. (%)	
	Placebo (n = 104)	Losartan (n = 101)
Location of enrollment		
ED	20 (19.2)	20 (19.8)
Floor	63 (60.6)	59 (58.4)
Step-down or intermediate care	12 (11.5)	5 (5.0)
ICU	9 (8.7)	17 (16.8)
Sex		
Men	63 (60.6)	60 (59.4)
Women	41 (39.4)	41 (40.6)
Age, y		
Mean (SD)	56.4 (15)	53.8 (16.1)
18-34	5 (4.8)	13 (12.9)
35-54	41 (39.4)	42 (41.6)
55-64	34 (32.7)	23 (22.8)
≥65	24 (23.1)	23 (22.8)
BMI		
Mean (SD)	33.1 (8.9)	34 (9.9)
<25	13 (12.6)	10 (9.9)
25 to <30	26 (25.2)	26 (25.7)
≥30		
30 to <35	36 (35)	30 (29.7)
35 to <40	7 (6.8)	16 (15.8)
≥40	21 (20.4)	19 (18.8)
Race^a^		
White	47 (45.2)	35 (34.7)
Black	30 (28.8)	37 (36.6)
Native American or Alaska Native	1 (1.0)	0
Asian	2 (1.9)	7 (6.9)
Hispanic	18 (17.3)	19 (18.8)
Other or unknown	6 (5.8)	3 (3.0)
Ethnicity		
Not Hispanic or Latino	76 (73.1)	80 (79.2)
Hispanic or Latino	23 (22.1)	20 (19.8)
Unknown	5 (4.8)	1 (1.0)
Insured^b^	80 (77.7)	83 (82.2)
Medicaid	18 (17.3)	18 (17.8)
Medicare	32 (30.8)	21 (20.8)
Private	42 (40.4)	59 (58.4)
Comorbidities		
Coronary artery disease	7 (6.7)	3 (3.1)
Hypertension	46 (44.2)	35 (34.7)
Using hypertension medication^c^	29 (63.0)	21 (60.0)
Congestive heart failure	4 (3.8)	1 (1.0)
Pulmonary hypertension	4 (3.9)	0
Asthma	14 (13.5)	13 (12.9)
COPD	10 (9.6)	10 (10.1)
Chronic bronchitis	2 (1.9)	0
Obstructive sleep apnea	20 (19.4)	6 (6.1)
Diabetes	26 (25.0)	19 (19.2)
Tobacco or nicotine user	9 (8.7)	4 (4)
Vital signs at enrollment		
Blood pressure, mm Hg		
Systolic	124.2 (18.1)	127.9 (16)
Diastolic	72 (11.5)	73.5 (12.3)
Temperature, ° C	37.6 (6.1)	36.9 (0.8)
Heart rate, bpm	81.1 (15.5)	81.6 (15.5)
Respiratory rate, breaths per min	21.3 (5.9)	20.5 (4.7)
Pulse oximetry, %	94.5 (2.5)	94.1 (2.8)
Baseline laboratory results, mean (SD)		
WBC, /μL	7300 (3900)	7200 (3600)
Platelets, ×10^3^/µL	227.8 (91.4)	221.4 (78.9)
Creatinine, mg/dL	0.8 (0.2)	0.9 (0.3)
Baseline pulmonary values		
Pao_2_:Fio_2_ ratio, median (IQR)	214.1 (138.4-253.4)	214.1 (153.6-258.4)
Type of supplemental o_2_ at enrollment		
None	10 (9.6)	18 (17.8)
Nasal cannula	57 (54.8)	58 (57.4)
Facemask or nonrebreather	11 (10.6)	2 (2.0)
CPAP	1 (1.0)	0
BIPAP	6 (5.8)	4 (4.0)
High-flow nasal cannula	12 (11.5)	14 (13.9)
Endotracheal intubation	7 (6.7)	6 (5.9)
ECMO	0	0

Abbreviations: BIPAP, bilevel positive airway pressure; BMI, body mass index (calculated as weight in kilograms divided by height in meters squared); bpm, beats per minute; COPD, chronic obstructive pulmonary disease; CPAP, continuous positive airway pressure; ECMO, extracorporeal membrane oxygenation; Pao_2_:Fio_2_, arterial partial pressure of oxygen to fraction of inspired oxygen; WBC, white blood cells.

SI conversion factors: To convert creatinine to micromoles per liter, multiply by 88.4; platelets to ×10^9^/L, multiply by 1; WBC to ×10^9^/L, multiply by 0.001.

^a^ Individuals who reported Hispanic as their race did not report White or non-White Hispanic ethnicity. Other race includes American Indian, multiple races, and those who chose not to answer.

^b^ Totals may add to greater than 100% owing to multiple insurance categories.

^c^ Data were missing for 58 participants (56%) in the placebo group and 66 participants (65%) in the losartan group. No other variable missing more than 3 participants.

Primary outcome data were missing in 16 participants (15.4%) in the placebo group and 10 participants (9.9%) in the losartan group, owing to an inability to contact the participant on day 7 or participant failure to record pulse oximetry values. This difference reflects a higher proportion of participants discharged prior to day 7 in the placebo group than the losartan group (62 participants [59.6%] vs 49 participants [48.5%]). Three participants (2.9%) in the placebo group and 2 participant (2.0%) in the intervention arm died prior to day 7.

**Figure 2.**
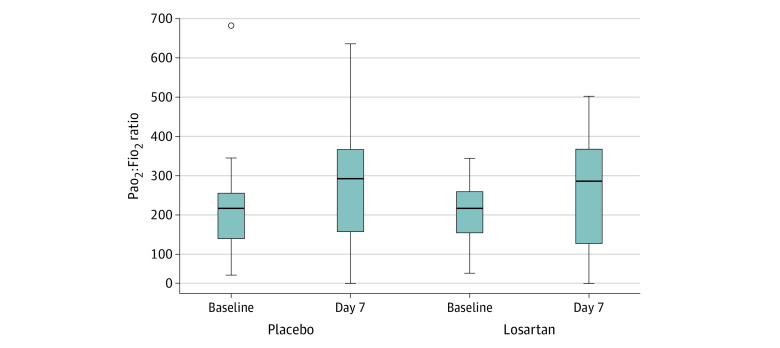
Arterial Partial Pressure of Oxygen to Fraction of Inspired Oxygen (Pao_2_:Fio_2_) Ratios for Losartan and Placebo Groups

Based on raw data, Pao_2_:Fio_2_ ratio did not differ between the placebo and intervention groups (median [IQR], 297 [196-366] vs 297 [130-366]) (Figure 2). In our primary analysis, the estimated effect of losartan on the Pao_2_:Fio_2_ ratio, expressed as mean difference between groups, was −24.8 (95% CI, −55.6 to 6.1; *P* = .12). Secondary analyses excluding early deaths resulted in an estimated effect of −27.1 (95% CI, −57.8 to 3.7; *P* = .08), while a complete case analysis yielded −23.6 (95% CI, −55.6 to 8.5; *P* = .15).

**Figure 3.**
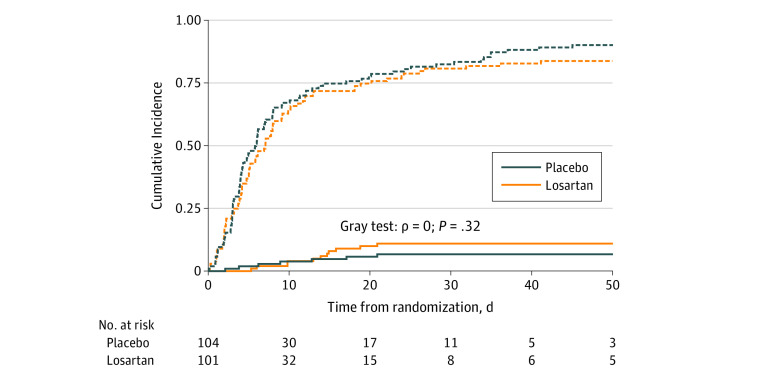
Cumulative Incidence of Death and Discharge in Losartan and Placebo Groups Solid lines indicate death; dotted lines, discharge.

We observed no difference in time to hospital discharge or in-hospital mortality (Figure 3). By day 28, 11 participants (10.6%) in the intervention group died vs 9 participants (8.9%) in the placebo group died, equilibrating to 11 participants in each group by day 90. There were no differences in freedom from o_2_ or mechanical ventilation in the first 10 days following randomization, and the ordinal outcome did not differ between treatment groups.

More patients in the losartan group than the placebo group required vasopressors (20 patients [20.0%] vs 11 patients [10.7%]; *P* = .08), contributing to fewer vasopressor-free days in the intervention group (median [IQR], 8.7 [8.2-9.3] vs 9.4 [9.1-9.8]; *P* = .04) (Table 2). Adverse events did not differ significantly by treatment allocation (eTable 2 in Supplement 2), although a numerically higher number of cardiovascular SAEs were noted in the losartan group. Acute kidney injury by Kidney Disease: Improving Global Outcomes (KDIGO) score was more common in the interventional group (20 patients [19.4%] vs 9 patients [8.8%]; *P* = .04).

**Table 2.** Alive and Intervention-Free Days Analyses

Intervention	No./No. (%)		*P* value
	Placebo	Losartan	
Required supplemental oxygen	94/104 (90.4)	89/101 (88.1)	.67
Alive and oxygen-free days through day 28, median (IQR)	18.4 (16.5-20.4)	18.1 (16.1-20.1)	.83
Required intubation	17/103 (16.5)	21/100 (21.0)	.47
Required second intubation	4/103 (3.9)	1/100 (1.0)	.37
Alive and ventilator-free days through day 28, median (IQR)	24.6 (23.1-26.1)	23.6 (21.8-25.3)	.38
Required vasopressors	11/103 (10.7)	20/100 (20.0)	.08
Alive and vasopressor-free days through day 10, median (IQR)	9.4 (9.1-9.8)	8.7 (8.2-9.3)	.04

Among 128 patients for whom viral load data were available, there was no difference in viral load by treatment group (eFigure 1 in Supplement 2). We detected no losartan or carboxylosartan in any placebo treated participant, while losartan and carboxylosartan pharmacokinetics were consistent with expected values in those treated with losartan (eFigure 2 and eTable 3 in Supplement 2). Analysis of RAAS biomarkers demonstrated no significant effect of losartan on the change in angiotensin-(1-7), AII, ACE, or ACE2 over time in 55 patients with available samples (eTable 4, eFigure 3, and eFigure 4 in Supplement 2).

## Discussion

In this multicenter, blinded, and placebo-controlled randomized clinical trial, 205 hospitalized participants with COVID-19 and acute lung injury not already using RAAS inhibitors were randomized to oral losartan at the maximum dose approved by the US Food and Drug Administration to test the hypothesis that AT1R blockade improves pulmonary function. Despite a moderate to severely ill cohort, well-matched baseline Pao_2_:Fio_2_ ratios, and pharmacologically relevant steady state concentrations, we found no evidence that treatment with losartan improved lung injury or clinical outcomes, nor significantly affected major circulating RAAS components.

It is important to note that we observed 2 potentially harmful effects of losartan on hemodynamics and kidney function. However, a higher proportion of participants in the intervention group were enrolled in the ICU, indicating potential small imbalances in severity of illness at enrollment. Generally, patients requiring the ICU are characterized by older age, are more likely to be a race other than White and to be men, and have more comorbidities.^34^ These patients may be more likely to exhibit cytokine storm related to inflammaging^35^ or baseline metabolic syndrome, although these factors were well matched by randomization, making them less likely to contribute to initial disposition.

Not surprisingly, based on the mechanism of action, patients in the losartan group were more likely to require vasopressor support. Furthermore, while the losartan group was more likely to meet acute kidney injury criteria by KDIGO, this finding may indicate expected physiologic changes in intraglomerular blood flow rather than actual tissue injury per se. Nevertheless, in the absence of benefit and potential for harm, we conclude losartan treatment is not indicated in this setting.

In light of our results, it is important to consider the pharmacology and biochemical effects of the intervention. The observed pharmacokinetics of losartan and carboxylosartan are consistent with reported values^22^ and maximal AT1R blockade.^36^ As the half-lives of losartan and carboxylosartan are approximately 2 and 6 hours, and time to maximal concentrations were 1 and 4 hours, respectively, steady state was achieved in the first day of dosing. However, plasma AII and angiotensin-(1-7) concentrations and serum ACE and ACE2 activities remained unaffected. Potential explanations for this finding include a narrow intervention window without sufficient time to yield significant effects, poor specificity of circulatory RAAS (unknown tissue source), or relative lack of RAAS dysregulation at baseline in this population,^4^ as more recent observations suggest SARS-CoV-2 does not necessarily induce unique, clinically relevant RAAS alterations, even in patients who develop severe disease. These observations regarding the lack of effect on the RAAS system are consistent with the lack of observed effect on the clinical outcomes. However, future analyses could investigate whether the circulatory RAAS is a mediator for treatment-induced effects on the outcomes.^37^

These results stand in contrast to an open-label trial of telmisartan that demonstrated significantly reduced 30-day mortality in the treatment group compared with placebo (4.3% vs 22.5%).^38^ While the differences could be explained by intraclass differences in the agents, it should be noted the study by Duarte et al^38^ was designed to detect a reduction in C-reactive protein, was unblinded, excluded ICU patients, and was terminated early owing to slow recruitment, making comparison difficult. Meanwhile, 2 additional trials examined the effects of discontinuation vs continuation of RAAS inhibitors on admission for COVID-19. These studies found no significant differences between continuation and discontinuation groups, consistent with the findings of our trial.^39,40^ Additionally, we recently published our experience in an analogous trial in the outpatient setting.^29^ Although that trial was terminated early, it found that losartan did not affect hospital admission, dyspnea, or quality of life. Given the relatively healthy cohort enrolled in that trial, it remains unclear whether more severely ill patients might benefit from losartan. However, the results of this study do not support the hypothesis that losartan effectively mitigates viral-induced acute lung injury in COVID-19, with uncertain implications for other, potentially more potent, agents that target the RAAS. This study further contributes to the literature in this field.

### Limitations

There are several limitations to consider with this study. The trial was initiated early in the pandemic, and temporal changes in clinical care, including introduction of dexamethasone and remdesivir, may have biased the trial toward the null. Our findings may also be consistent with the hypothesis that the RAAS does not play a significant role in SARS-CoV-2–related acute lung injury relative to other inflammatory pathways. Our choice of primary efficacy outcome required differential imputation methods based on whether or not the participant was treated with positive pressure ventilation, potentially affecting the results. However, the lack of effect on o_2_- or mechanical ventilation–free days decreases the likelihood that a larger study would identify clinically meaningful effects. While we detected sufficient concentrations of losartan, it remains possible lung parenchymal penetration is limited, mitigating potential efficacy. The relatively small subgroup undergoing RAAS measurement may have been underpowered to detect treatment effects. It remains possible that certain subgroups may benefit from ARB treatment that we could not identify owing to inadequate subgroup sample size, including patients who may have RAAS dysregulation at baseline prior to infection. Additionally, we cannot necessarily generalize these findings to other ARBs, ACEIs, or other agents that modulate the RAAS.

## Conclusions

This randomized clinical trial found that initiation of oral losartan to hospitalized patients with COVID-19 and acute lung injury does not improve Pao_2_:Fio_2_ ratio at 7 days. These data have implications for ongoing clinical trials.

## Notes

Supplement 1.
Trial Protocol and Statistical Analysis Plan

Supplement 2. **eTable 1.** Pao_2_:Fio_2_ Ratio at Baseline With Day 7 Results **eTable 2.** Inpatient Nonfatal Adverse Events Per Person-Day, Days 1-15 **eTable 3.** Losartan and Carboxylosartan Pharmacokinetic Characteristics **eTable 4.** Association Between Treatment Group and Circulatory RAAS Components After Adjusting for Baseline and Time and Jointly Modeling the Outcome and Time-to-Discharge **eFigure 1.** Effect of Losartan on Relative Viral Load **eFigure 2.** Losartan and Carboxylosartan Concentrations **eFigure 3.** Plasma Log(AII) From Days 1-15, by Treatment Allocation **eFigure 4.** Plasma Log(angiotensin-[1-7]) From Days 1-15, by Treatment Allocation

Supplement 3.
Angiotensin Receptor Blocker Based Lung Protective Strategies for Inpatients With COVID-19 (ALPS-IP) Investigators

Supplement 4.
Data Sharing Statement
