# Error in Nonauthor Collaborators

**DOI:** 10.1001/jamanetworkopen.2022.15958

**Published:** 2022-05-17

In the Original Investigation titled “Efficacy of Losartan in Hospitalized Patients With COVID-19–Induced Lung Injury: A Randomized Clinical Trial,”^1^ published March 16, 2022, Nastasia James was missing from the list of Angiotensin Receptor Blocker Based Lung Protective Strategies for Inpatients With COVID-19 (ALPS-IP) Investigators. This article has been corrected.^1^
