# Supplementary material for: Efficacy of Losartan in Hospitalized Patients With COVID-19–Induced Lung Injury: A Randomized Clinical Trial
Source: JAMA Netw Open. 2022 Mar 16;5(3):e222735. doi: 10.1001/jamanetworkopen.2022.2735 (PMC8928006; doi:10.1001/jamanetworkopen.2022.2735)
Supplement: Supplement 1. — Trial Protocol and Statistical Analysis Plan [file jamanetwopen-e222735-s001.pdf]

**Protocol Title**	Randomized Controlled Trial of Losartan for inpatients with COVID-19
**Principal Investigator/Faculty Advisor -1**	Name: Christopher Tignanelli, MD
	Department: Surgery
	Telephone Number:612 625 7911
	Email Address: ctignane@umn.edu
**Principal Investigator/Faculty Advisor -2**	Name: Michael Puskarich, MD, MS
	Current Academic Status (Student, Fellow, Resident): NA
	Department: Emergency Medicine
	Telephone Number: 612 626 6911
	Email Address: mike-em@umn.edu
**Scientific Assessment**	HRPP facilitated scientific assessment
**IND/IDE # (if applicable)**	148365
**IND/IDE Holder**	Michael Puskarich, MD MS
**Investigational Drug Services # (if applicable)**	NA
**Version Number/Date:**	Version: 2.1<br>Date: 16DEC2020

Co-Investigators:
Jeffrey Chipman, MD
Department: Surgery
Title: Professor and Chief, Division of Critical Care / Acute Care Surgery
M Health Fairview Critical Care Domain Lead
Email: chipm001@umn.edu

Timothy Shacker, MD
Department: Medicine and Infectious Diseases
Title: Vice Dean for Research, University of Minnesota Medical School
Director, Program in HIV Medicine
Email: schac008@umn.edu

Nicholas Ingraham, MD
Department: Medicine
Title: Pulmonary Critical Care, Fellow
Email: ingra107@umn.edu

David Wacker, MD
Department: Medicine
Title: Assistant Professor of Medicine
Email: wack0012@umn.edu

Ron Reilkoff, MD

Department: Medicine
Title: Assistant Professor of Medicine, Medical Director M Health Fairview ICU
Email: rreilkoff@umn.edu

Michelle Biros, MD, MS
Department: Emergency Medicine
Title: Professor and Research Director, Department of Emergency Medicine; Deputy Institutional Official, University of Minnesota Biomedical Research
Email: biros001@umn.edu

John Connett, PhD
Department: Biostatistics
Title: Professor of Biostatistics and Director of CTSI Biostatistical Design and Analysis Center (BDAC)
Email: john-c@umn.edu

Tamara Bezdicek, PharmD
Department: Pharmacy
Title: Clinical Manager at Fairview Southdale
Email: tsobask1@fairview.org

REVISION HISTORY

Revision #	Version Date	Summary of Changes	Consent Change?
1.7	3/26/20	Changes to I/E criteria, method of patient identification, data collected, and corrected inconsistencies. Additional sites added.	Y – updated biospecimens and risks
1.8	04/07/20	Updated I/E, change in primary efficacy outcome measurement, increased dose, updated optional PK substudy, decreased frequency of blood draws from daily to every other day, clarified table of study procedures	Y – updated optional PK substudy
1.9	04/13/20	Updated enrollment window to 48 hours, new language regarding compensation for study-related damages due to recent federal government issued orders, clarified conflicting exclusion criteria and protocol inconsistencies	Yes
2.0	04/28/2020	Updated approved viral swab methods to include oropharyngeal (OP) swabs, clarification of P/F ratio calculations for subjects discharged prior to Day 7	Yes
2.1	12/16/2020	Updated Covid symptoms in light of new evidence and clarified bioassays. Clarified DSMB schedule.	No

ABBREVIATIONS/DEFINITIONS

- COVID-19 Disease caused by the virus SARS-CoV-2, or “Wuhan coronavirus”
- ACE2 Angiotensin converting enzyme 2
- ACE Angiotensin converting enzyme
- ACEi Angiotensin converting enzyme inhibitor
- AE Adverse event
- ALI Acute lung injury
- ARDS Acute Respiratory Distress Syndrome
- ARF Acute respiratory failure
- ARB Angiotensin receptor blocker
- AT1R Angiotensin 1 receptor
- CDR Clinical Data Repository
- CDSS Clinical decision support systems
- CTSI Clinical and Translational Science Institute
- D & I Dissemination and Implementation
- DUA Data Use Agreement
- EBP Evidence-based best practice
- eConsent Electronic consent
- ECMO Extracorporeal membrane oxygenation
- ED Emergency department
- EHR Electronic health record
- FiO2 Fraction of inspired oxygen
- ICD International Classification of Diseases
- ICF Informed Consent Form
- ICS Informatics Consulting Service
- ICU Intensive care unit
- LOS Length of stay
- NETT Neurological Emergencies Treatment Trials
- PaO2 Partial pressure of arterial oxygen
- SAE Severe adverse event
- PETAL Prevention and Early Treatment of Acute Lung injury
- SaO2 Saturation of oxygen
- SIREN Strategies to Innovate Clinical Trials Network
- SOFA Sequential organ failure assessment
- UMMC University of Minnesota Medical Center

Table of Contents

1.0	Objectives	8
2.0	Background	11
3.0	Study Endpoints/Events/Outcomes	15
4.0	Study Intervention(s)/Investigational Agent(s)	17
5.0	Procedures Involved	18
6.0	Data and Specimen Banking	21
7.0	Sharing of Results with Participants	23
8.0	Study Population	24
10.0	Local Number of Participants	32
11.0	Local Recruitment Methods	32
12.0	Withdrawal of Participants	33
13.0	Risks to Participants	34
14.0	Potential Benefits to Participants	35
15.0	Statistical Considerations	35
16.0	Health Information and Privacy Compliance	36
17.0	Confidentiality	40
18.0	Provisions to Monitor the Data to Ensure the Safety of Participants	40
19.0	Provisions to Protect the Privacy Interests of Participants	41
20.0	Compensation for Research-Related Injury	42
21.0	Consent Process	42
22.0	Setting	43
23.0	Multi-Site Research	44
24.0	Coordinating Center Research	45
25.0	Resources Available	45
26.0	References	45

1.0 Objectives

1.1 *Title*: Randomized Controlled Trial of Losartan for inpatients with COVID-19

1.2 *Purpose:* To determine the impact of early losartan compared to placebo on the change in respiratory failure in inpatients diagnosed with COVID19 requiring hospital admission at the time of initial presentation.

1.3 *Hypothesis:* Losartan will significantly improve respiratory failure, as measured by change in estimated (PEEP adjusted) P/F ratio, in patients requiring hospitalization.

1.4 *Study design:* Multi-center, prospective, randomized blinded interventional trial of losartan (50 mg orally twice daily) versus placebo administered for 10 days or until hospital discharge.

1.5 *Study procedures:*

- The trial emphasizes early intervention to prevent the progression of respiratory disease. Patients presenting to the emergency department (or transferred) who test positive for Covid-19 will be eligible for enrollment and randomization within 48 hours of admission to the hospital *or* within 48 hours of a positive test result, whichever is later. Patients transferred from another facility will be eligible.
- Investigators and research coordinators will leverage already functional electronic consent procedures to enroll patients safely while also minimizing risk to study staff. The telemedicine and eConsent platforms to be leveraged in this trial are actively used in NIH supported StrokeNet clinical trials, and are HIPAA compliant. If unable to utilize eConsent, paper consent will be utilized and a scanned copy uploaded into eConsent platform in REDcap.
- Enrolled patients will be randomized to losartan or placebo, and undergo daily assessment by study personnel with built-in safety and efficacy assessments. Enrolled participants will be administered study drug twice daily while hospitalized. Study personnel will evaluate patients every day during the intervention window to review symptoms for possible adverse events and administer validated patient-reported dyspnea assessment at predetermined time points. If in-person examinations are necessary, study investigators trained for appropriate PPE use will perform the examinations.
- Nasopharyngeal or oropharyngeal swabs and blood samples will be collected from the same nostril and transported to a BSL-3 laboratory (https://www.researchservices.umn.edu/services-name/biosafety-level-3-program). These will be stored for public health and viral research purposes.
- Patients will be followed for 90 days to determine outcomes and review delayed adverse events. Follow-up phone contact and electronic medical record review will determine the primary and secondary efficacy endpoints, and provide additional safety data.

1.6 *Data and specimen collection method*

- Inclusion and exclusion criteria, patient demographics, baseline comorbidities, symptoms, and validated symptom assessment measurements by patient self-report (if patient is able) will be collected at the time of enrollment and maintained in a RedCap database. In addition, electronic consent forms will be maintained in a University of Minnesota RedCap database with established functionality for this purpose in our group. The database, with the currently approved eConsent policy, is HIPAA compliant.
- Daily follow-ups will be recorded by study personnel into a RedCap database and will include data points from the electronic medical record, and self-reported AEs. At enrollment and on day 4 and 10, standardized patient-reported dyspnea and SF-12 surveys will be administered. In the event the patient is not available or unable to participate on the day of survey administration, surveys administered +/- 1 day will be considered before this data point is considered missing. If the patient is unable to participate, this will be recorded. The following data points will also be collected, if available:
    - Name
    - Date of birth
    - Medical record number
    - Phone number
    - Email address
    - Age
    - Gender
    - Race
    - Ethnicity
    - Insurance status (if available)
    - Zip code
    - Location of initial sample collection for diagnosis
    - Location of enrollment
    - Date and time of initial sample collection for diagnosis
    - Date and time of positive test result for Covid-19
    - Date of first symptoms
    - Date of first fever (temperature >101.5)
    - Vital signs at presentation and daily
    - Comorbidities [hypertension (requiring or not requiring medication), diabetes mellitus (insulin or non-insulin dependent), coronary artery disease, myocardial infarction, congestive heart failure (with preserved or reduced ejection fraction, if known), pacemaker or AICD, asthma (with or without emergency department evaluation and / or hospital admission in the past year), chronic obstructive pulmonary disease, chronic bronchitis, chronic steroid use, history of transplant (with type), arrhythmias including atrial fibrillation, dialysis, angina, pulmonary hypertension and obstructive sleep apnea, renal disease, liver disease, tobacco and alcohol use history, recent pregnancy or breastfeeding history, height and weight]
    - Home medications [antihypertensives, insulin, non-insulin diabetes medications, corticosteroids (inhaled or systemic), other

immunosuppressants, Use of outpatient COVID-directed treatments (such as hydroxychloroquine, Tylenol, ibuprofen, azithromycin, or others) or home oxygen use
  - Concurrent inpatient medications, including COVID-directed treatments, as well as study medication administration timing
  - PROMIS Pool v1.0 Dyspnea Characteristics Survey (5 question Likert scale survey) (attached) with date/time of administration
  - Short form 12 health survey (attached) with date/time of administration
  - Nasopharyngeal or oropharyngeal swab every third day (performed, yes or no, reason if not)
  - Research blood samples (performed, yes or no, reason if not)
  - Hospital and ICU utilization (with discharge date, if applicable)
  - Clinical laboratory results daily for 10 days, if available (creatinine, total bilirubin, platelet count, white blood count, hemoglobin, sodium, potassium, chloride, glucose, lactate, procalcitonin, albumin, AST, ALT, partial pressure of oxygen on arterial blood gas, troponin, BNP or proBNP, urine pregnancy test, influenza or viral testing results)
  - Radiographic findings of CXR, CT chest
  - EKG QTc (if performed)
  - Ventilatory support: type of ventilatory support (nasal cannula, high flow nasal cannula, face mask, CPAP, BiPAP, endotracheal intubation, ECMO), fraction of inspired oxygen, oxygen flow rate (if applicable), ventilator settings (if applicable, including tidal volume and PEEP)
  - Vasopressor usage (daily type and dose)
  - Need for renal replacement therapy
  - Evidence of bacterial co-infection
  - Glasgow coma scale (daily, if available)
  - Death (with date, if applicable)
  - Study withdrawal, with date and time (if applicable)

- Final outcome follow-ups will be determined through a combination of patient report, electronic medical record review, and review of the social security death index (SSDI).

- All data will be stored in a password protected Redcap database, with customizable tiered access and an audit trail. All protected health information (PHI) will be marked with a PHI tag that will prevent export, so only deidentified data can be exported from the database. Only deidentified data with no PHI will be exported for analysis.

- Nasopharyngeal or oropharyngeal swabs (day 1,4,8,10,15); 30 patients will receive blood draws for pharmacokinetic analysis (day 1, hours 2, 4, and 6) and patients will provide blood samples for RAAS, RNASeq, epigenetic analysis of human genomes, flow cytometry, and cytokine assays, collected by study personnel or bedside nurse and placed in pre-packaged transport containers to be collected by biosafety-trained transporters, transported and processed to the University of Minnesota's BSL-3 laboratory noted above, and frozen at -80 C until later analysis for

local sites. Blood samples will not exceed 30 mL day 1, and will equal ~21-24 mL on days 2, 4, 6, 8, 10, and 15). All biospecimens will be labeled with a deidentified patient ID number to prevent inadvertent disclosure of PHI. Participants will be given the opportunity to contribute to an optional study using genetic material for future studies involving human genetic sequencing and other analysis of patient-level responses to COVID-19. These samples will likewise be deidentified, but are theoretically identifiable biologic information.

- Cytokine assays will be conducted by Timothy Schacker's laboratory at the University of Minnesota
- RAAS pathway assays will be conducted at the Wake Forest Hypertension and Vascular Research Biomarker Analytical Core
- Pharmacokinetics will be conducted at Courtney Fletcher's Antiviral Pharmacology Laboratory at the University of Nebraska

2.0 Background

1.2 Significance of Research Question/Purpose:

Since appearing in Wuhan, China in December 2019, the severe acute respiratory syndrome coronavirus 2 (SARS-CoV-2) has caused disease (COVID-19) in over 800,000 people with over 40,000 deaths to date across 180 countries.[1-3] The mortality is estimated at 3.7%,[4] with infectivity (R0) estimated at 2.24 to 3.585,[5] with morbidity and mortality disproportionately affecting the elderly.[6] Most severely affected patients are admitted for respiratory support and die of respiratory failure.[7,8] At present there is no current specific treatment for patients with COVID-19.[9] The novel coronavirus is closely related to the severe acute respiratory syndrome coronavirus (SARS-CoV) which caused an outbreak of disease (SARS) in 2003; this may inform the development of novel treatments.[9,10] Attempts to develop vaccines since SARS or middle eastern respiratory virus (MERS) have been unsuccessful, will take time, and may or may not be effective for this or future coronavirus pandemics.[11] Extensive efforts to date, therefore, have been appropriately focused on screening, identification, and containment with collaboration and coordination within and between governments to combat its spread.[12]

Due to the nature of coronavirus cellular binding, there exist several promising novel pharmacotherapeutic strategies that differentiate this virus from seasonal or pandemic influenza. Similar to the 2002-2003 SARS-CoV, the SARS-CoV-2 virus binds the spike protein of angiotensin-converting enzyme 2 (ACE2), a critical component of the renin-angiotensin-aldosterone (RAAS) system, in order to enter the cell.[13] **(Figure 1 – red arrow on right)** Multiple proposed interventions to combat SARS-CoV-2 under development focus on this pathway with the goal of blocking viral entry into cells, and include anti-viral agents,[14] anti-malarial medications,[15] vaccines,[16] and viral protein inhibitors.[9] While these therapies are promising, immediate use of these interventions are not feasible. In many cases, drugs are still under development and lack a clinical indication, with limited safety and efficacy data in humans to date. These factors will necessarily lead to delays in drug development. Given the rapid spread of SARS-CoV-2, patients would greatly benefit from interventions with a well-established safety profile readily available for immediate clinical trial testing and rapid knowledge translation if efficacious.

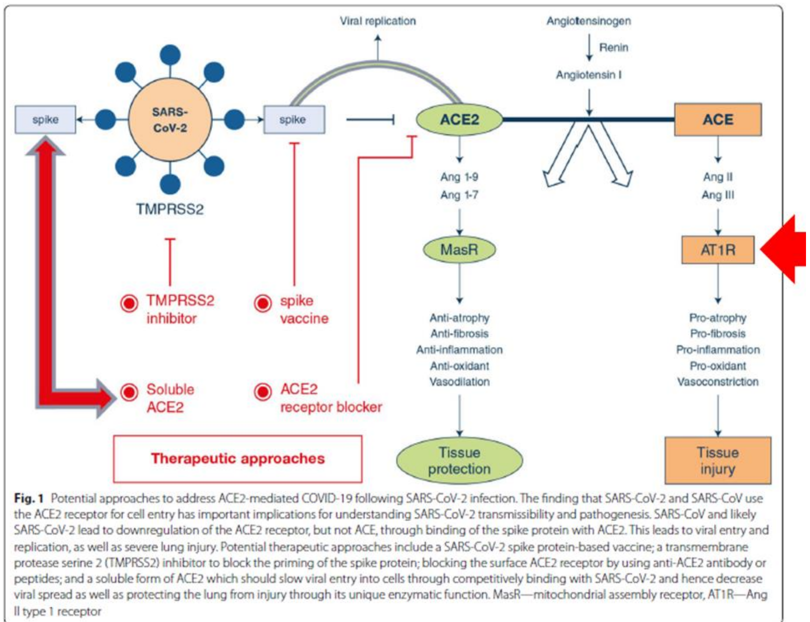

Fig. 1 Potential approaches to address ACE2-mediated COVID-19 following SARS-CoV-2 infection. The finding that SARS-CoV-2 and SARS-CoV use the ACE2 receptor for cell entry has important implications for understanding SARS-CoV-2 transmissibility and pathogenesis. SARS-CoV and likely SARS-CoV-2 lead to downregulation of the ACE2 receptor, but not ACE, through binding of the spike protein with ACE2. This leads to viral entry and replication, as well as severe lung injury. Potential therapeutic approaches include a SARS-CoV-2 spike protein-based vaccine; a transmembrane protease serine 2 (TMPRSS2) inhibitor to block the priming of the spike protein; blocking the surface ACE2 receptor by using anti-ACE2 antibody or peptides; and a soluble form of ACE2 which should slow viral entry into cells through competitively binding with SARS-CoV-2 and hence decrease viral spread as well as protecting the lung from injury through its unique enzymatic function. MasR—mitochondrial assembly receptor, AT1R—Ang II type 1 receptor

***Figure 1**: Mechanisms of Action of SARS-CoV-2 interaction with the Renin Aldosterone Angiotensin System and possible therapeutic options proposed by Zhang et al.[9]*

1.3 Rationale and Scientific Hypothesis:

Renin-aldosterone blockade has been proposed as a potential treatment for COVID-19.[17-19] This hypothesis was originally published by Sun et al[19] on February 16, 2020 (manuscript published in Chinese) and in the <u>British Medical Journal</u> on February 4, 2020.[18] This hypothesis was reinforced in <u>Drug Development Research</u> on March 4, 2020.[17] To the best of our knowledge, our study is the first investigating this hypothesis.

As opposed to ongoing clinical trials targeting viral binding and entry, the proposed clinical trial proposes to mitigate the respiratory consequences of SARS-CoV-2, thereby reducing morbidity and mortality. As illustrated in Figure 1, SARS-CoV-2 binds to angiotensin converting enzyme 2 (ACE2), similar to 2002-2003 SARS-CoV. Molecular simulation has shown SARS-CoV-2 has a higher affinity for ACE2 compared with SARS-CoV.[20]

<u>Healthy State:</u>

In states of health, ACE2 converts Angiotensin 2 (ATII) into lung-protective angiotensin (AT) 1-7 and 1-9, thus preventing unopposed ATII activation of AT1R. Unopposed AT1R activation triggers a downstream pro-inflammatory cascade, characterized by increased lung permeability, acute lung injury (ALI), acute respiratory distress syndrome (ARDS), and death **(Figure 2).**[4,18] AT1R activation also leads to type II pneumocyte apoptosis, a

common feature of ARDS.[12,13] Therefore, under usual conditions of health, ACE2 prevents lung injury.

Infection with SARS-CoV-2:

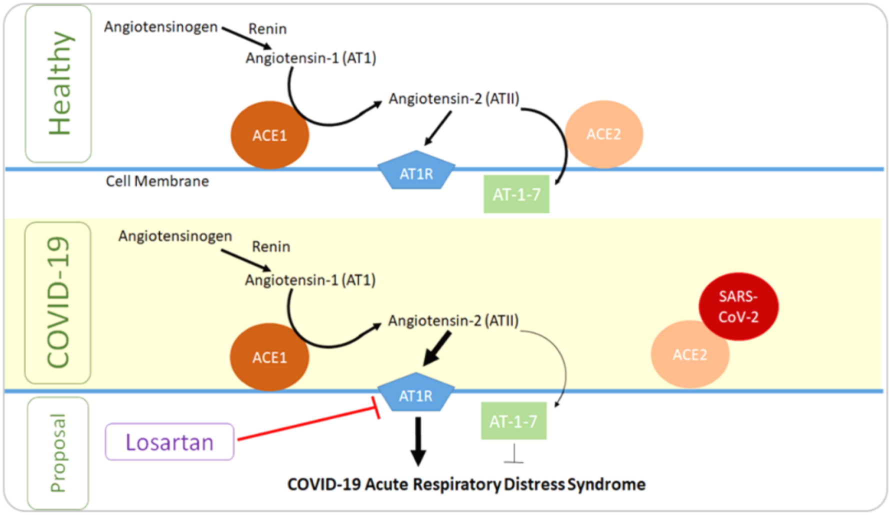

Figure 2: Proposed mechanism of Losartan in disrupting the pro-inflammatory and injurious pathway caused by over stimulation of AT1R in the setting of SARS-CoV-2 infection. ACE1-Angiotensin converting enzyme 1, AT1R-Angiotensin I receptor, ACE2-Angiotensin converting enzyme 2, AT1-7-Angiotensin 1-7.

Impaired ACE2 activity due to binding with SARS-CoV-2 results in excessive ATII allowing unopposed binding of AT1R without generation of lung-protective AT1-7 and AT1-9 **(Figure 2)**.[21,22] An ongoing clinical trial is attempting to exploit this mechanism by treating 24 patients with recombinant human ACE2 (rhACE2) infusions.[23] Infusions of ACE2 have previously been shown to result in decreased AT2 and increased AT1-7.[24] Unfortunately, protein recombinant human infusions are costly and scaling production will take significant time.

2.1 Given this, we propose to leverage a readily available angiotensin receptor blocker (ARB) with an excellent safety profile, losartan, to mitigate this pathway with the aim of decreasing ALI/ARDS. While ACE inhibitors (ACEi) could also be considered for trial, ACEi are frequently associated with cough which could exacerbate the spread of the disease. In 2 8 week studies, studies, losartan had 17% and 27% rates of cough vs 69% and 62% (see package insert). Furthermore, ACE inhibitors can induce expression of ACE2 secondary to low local angiotensin II levels, potentially increasing viral uptake.

Preclinical Supporting Data:

SARS-CoV specific preclinical work:
In 2003, ACE2 was identified as the binding protein for SARS-CoV (Li et al, Nature, 2003).[25] This was validated in 2005 by Kuba et al. This same study identified that SARS-CoV injury worsens acute lung injury and can be attenuated by Renin-Angiotensin (RAS) pathway inhibition (Kuba et al, Nature Med, 2005).[22] Kuba first identified that ACE2 knockout mice had significantly lower viral loads in the lung following infection, whereas wildtype (wt) mice had very high viral loads.[22] Additionally, wt mice had significantly higher lung injury scores following SARS-CoV infection.[22] SARS-CoV infection results in downregulation of its binding protein ACE2 (ACE2 has been shown to be lung protective in its action of converting AT2 -> AT1-7) (Kuba et al, Nature Med, 2005).[22] This finding that SARS-CoV results in downregulation of ACE2 has been validated in subsequent studies (Glowacka et al, Journal of Virology, 2010).[26] Kuba et al, then showed that treatment with Losartan 15 mg/kg attenuated lung injury (**Figure 3B)** and the development of pulmonary edema **(Figure 3C)** in mice infected with SARS-CoV Spike-FC.[22] For this study 2.5-3 month old mice received SARS-CoV Spike-FC. 30 minutes later they received

HCL to expedite lung injury. Mice were then randomized to Losartan vs vehicle. 1 and 2 hours later SARS-CoV Spike-FC. Mice then received 3 hours of mechanical ventilation and were sacrificed. **(Figures 3B and C)** (Kuba et al, Nature Med, 2005).[22]

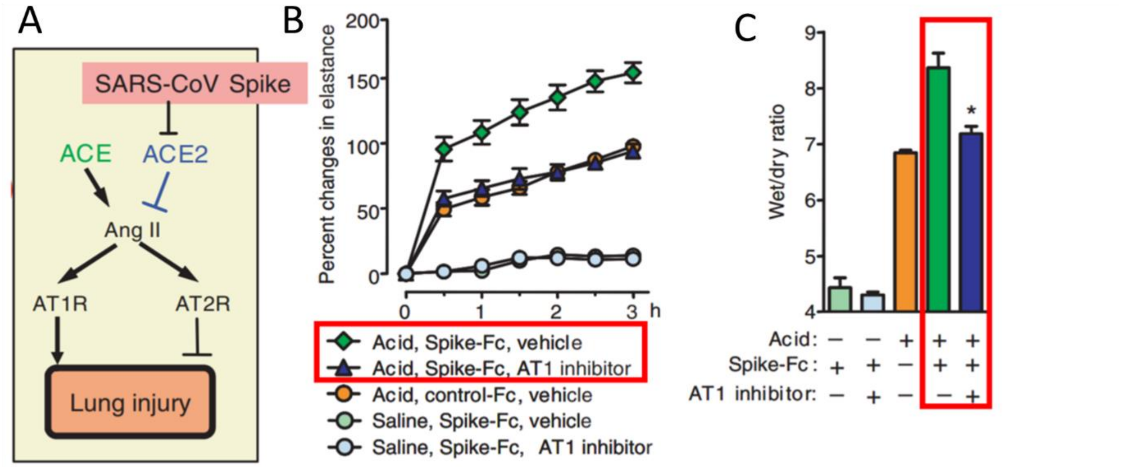

Figure 3: (A) Mechanism of action proposed by Kuba et al. (B) Lung injury in mice infected with SARS-CoV Spike-FC (green diamond) treated with vehicle compared with mice infected with SARS-CoV Spike-FC treated with Angiotensin-II receptor inhibitor (Losartan) (C) Lung wet/dry ratio for mice infected with SARS-CoV Spike-FC treated with vehicle vs. Angiotensin II receptor inhibitor (Losartan).
Figures from Kuba et al, Nature Med, 2005

In 2005, Imai et al showed that loss of ACE2 leads to significantly worse oxygenation, massive lung edema, and inflammatory cell infiltration in mice compared to wildtype mice in response to acid aspiration.[21] They then performed a rescue experiment and showed that supplementation with rhACE2 rescued infected mice **(Figure 4A, B: green circles vs purple triangle and dark pink diamond)**.[21]

Imai et al. demonstrated inhibition of AT1R via losartan reduced lung injury **(Figure 5, light purple triangle)** compared with vehicle **(Figure 5, purple square)**. This pathway was specific to inhibition of AT1R as, inhibition of AT2R did not reduce lung injury

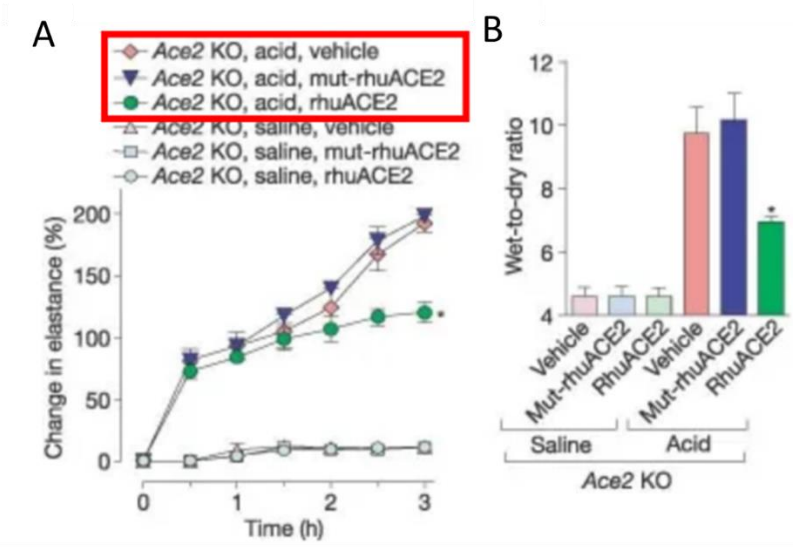

Figure 4: (A) ACE2 knockout (KO) mice treated with recombinant ACE2 (rhuACE2) verses vehicle or mutant rhuACE showed reduced lung injury (B) ACE2 knockout (KO) mice treated with recombinant ACE2 (rhuACE2) verses vehicle or mutant rhuACE showed reduced wet/dry ratio
Figures from Imai et al, Nature, 2005

Imai et al. then showed that inhibition of AT1R via losartan reduced lung injury **(Figure 5, light purple triangle)** compared with vehicle **(Figure 5, purple square)**. This pathway was specific to inhibition of AT1R as, inhibition of AT2R did not reduce lung injury **(Figure 5, pink triangles)** For this study ACE2

knockout (KO) mice were given AT1R (losartan) or AT2R 30 minutes prior to acid-induced acute respiratory distress syndrome (ARDS).[21]

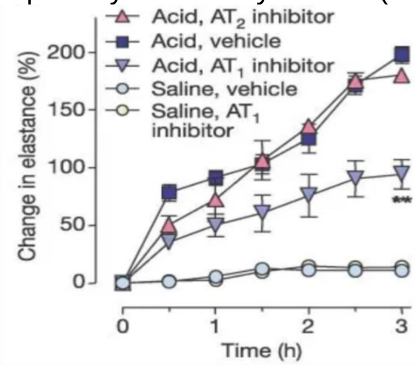

Figure 5: (A) ACE2 knockout (KO) mice treated with acid-induced ARDS treated with AT2R inhibition (pink triangle), AT1R inhibition (light purple triangle), or vehicle (purple square). *Figures from Imai et al, Nature, 2005*

<u>Preclinical supporting data for Renin-Angiotensin inhibition in other viral pneumonias that interact with ACE2:</u>

Both influenza H7N9 and H5N1 have been implicated to cause lung injury through ACE2 with resultant upregulation of angiotensin-2 and AT1R induced lung injury (Zou et al., Nature Communications, 2014 and Yang et al, Sci Rep, 2014).[27,28] Yiwu et al found that Losartan at clinically equivalent doses to humans was associated with increased survival, decreased lung edema and lung injury scores, and decreased IL-6 for mice infected with influenza H5N1 **(Figure 6 A-E)** (Yiwu et al, Sci China Life Sci, 2015).[29]

Yang et al infected 4 week old mice with either influenza H7N9 or placebo.[28] Mice were given Losartan 30 minutes prior to viral infection. They identified that inhibition of AT1R attenuates H7N9 lung injury and was associated with lower wet-dry ratios and lower infiltrative cell counts (Yang et al, Sci Rep, 2014).[28]

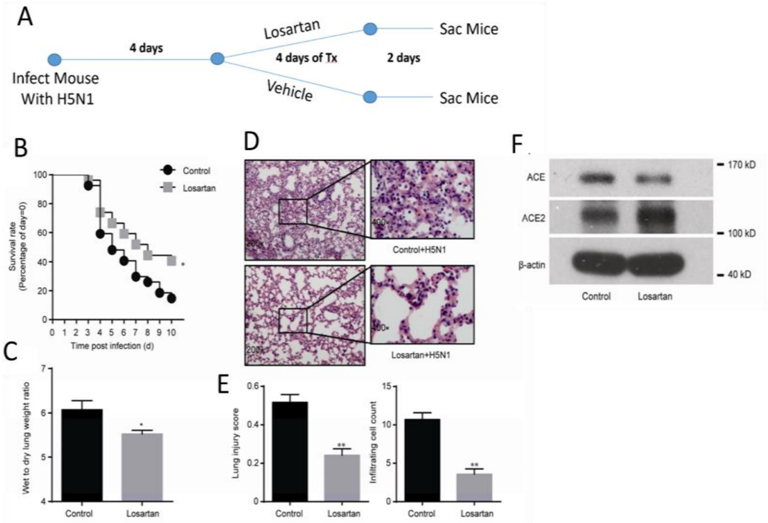

Figure 6: (A) Study Design (B) Mice infected with H5N1 had improved survival when treated with losartan verses control (C) Mice infected with H5N1 had less pulmonary edema when treated with losartan verses control (D) Mice infected with H5N1 had decreased pulmonary inflammation when treated with losartan verses control (E) Mice infected with H5N1 had reduced lung injury score when treated with losartan verses control (F) Mice infected with H5N1 had increased ACE2 (lung protective) and reduced ACE1 (lung deleterious) when treated with losartan verses control. *Figures from Yiwu et al, Sci China Life Sci, 2015*

2.2 **Existing Literature:**

There is no existing literature regarding the effect of losartan in COVID-19. **To that effect there is limited literature regarding the effectiveness of any treatments on COVID-19.** Data of relevance to are summarized in 2.1-2.3.

There is an urgency to identify effective and readily available treatments for patients with COVID-19. This study will elucidate the role of losartan, this is based on strong physiologic and preclinical data as described in detail above.

There is a critical and dire need to identify and evaluate all possible methods to minimize the burden of COVID-19 on our society and healthcare system. At the current rate of growth (as of 3/13/2020) America is 1 week from widespread outbreak similar to Italy and Iran. Unfortunately, current community efforts are now slowing down spread as our slope (see Figure below) is higher in the last few days than the rest of the world.

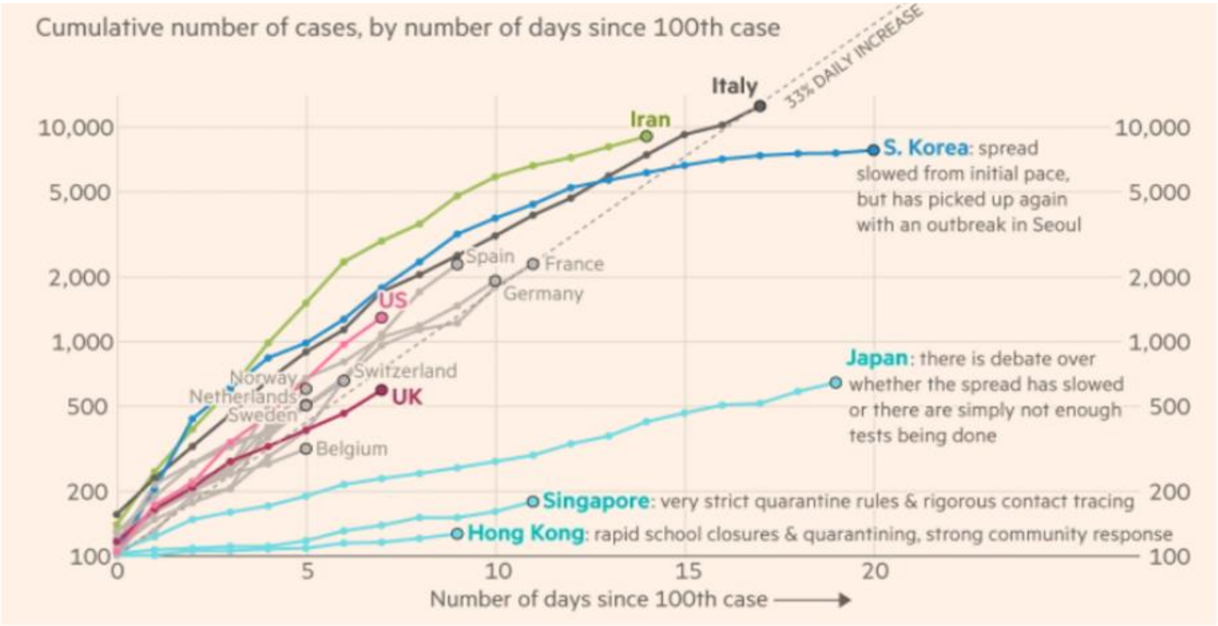

3.0 Study Endpoints/Events/Outcomes

3.1 *Primary Endpoint/Event/Outcomes:* The primary endpoint is to determine the impact of losartan on respiratory failure. This will be determined by measuring the difference in estimated (PEEP adjusted) P/F ratio at 7 days, a validated routinely used in critical care research derived from the partial pressure of oxygen or peripheral saturation of oxygen by pulse oximetry divided by the fraction of inspired oxygen (PaO2 or SaO2 : FiO2 ratio).[30,31] PaO2 is preferentially used if available. A correction is applied for endotracheal intubation and/or positive end-expiratory pressure. Patients discharged prior to day 7 will have a home pulse oximeter sent home for measurement of the day 7 value, and will be adjusted for home O2 use, if applicable. Patients who died will be applied a penalty with a P/F ratio of 0.

3.2 *Secondary Endpoint(s)/Event(s)/Outcome(s):*

3.3 There will be 7 secondary endpoints:

- To ensure losartan is safe and well tolerated in hospitalized patients with COVID-19, we will:
  - Compare daily hypotensive episodes (MAP < 65 mmHg) prompting intervention (indicated by a fluid bolus >=500 mL)
  - Hypotensive episodes requiring intervention (vasopressors or fluid bolus)
  - Determine the incidence of new or worsening renal failure

- Daily Sequential Organ Failure Assessment (SOFA) score to day 10. If data are unavailable for any given organ failure, we will use the last value carried forward approach.
- Change in estimated P/F ratio daily between groups
- In-hospital, 28, and 90-day mortality (by using state death certificate or social security death index data)
- Resource utilization as assessed by: ICU admission, ventilator free days, oxygen free days, vasopressor free days, ICU and hospital length of stay
- Respiratory failure as measured by the utilization of Bilevel Positive Airway Pressure (BiPAP), high flow nasal cannula, mechanical ventilation or extracorporeal membranous oxygenation (ECMO) utilization, as well as trends in S/F ratio.
- Symptomatic improvement as assessed by the dyspnea PROMIS and SF-12 assessments of dyspnea and functional status
- Percentage of subjects reporting each severity rating on the *7-point ordinal scale* [Time Frame: Day 15]
  - The *7-point ordinal scale* is an assessment of the clinical status at the first assessment of a given study day. The scale is as follows: 1) Death; 2) Hospitalized, on invasive mechanical ventilation or extracorporeal membrane oxygenation (ECMO); 3) Hospitalized, on non-invasive ventilation or high flow oxygen devices; 4) Hospitalized, requiring supplemental oxygen; 5) Hospitalized, not requiring supplemental oxygen; 6) Not hospitalized, limitation on activities; 7) Not hospitalized, no limitations on activities.
  - Change in viral load (nasopharyngeal or oropharyngeal swab and blood)

1.4 *Justification of the primary outcome:* This trial seeks to mitigate lung injury from Covid-19 rather than treating the viral infection. Most patients present to the hospital secondary to dyspnea and pneumonia, and the primary mechanism of death is via respiratory failure progressing to multi-system organ failure.[7] Prevention of progression of acute lung injury with an angiotensin receptor blocker such as losartan may decrease estimated P/F ratio, a validated measure of lung dysfunction that accounts for differences in the level of ventilatory support. We considered respiratory SOFA score, but this crude ordinal measurement is estimated off of the estimated P/F ratio, which will provide a more precise measurement of the same physiology. **Estimated P/F is a clinically relevant outcome not only to patients, but also to the healthcare system due to overwhelming surge of patients straining hospital resources which not only puts patients with COVID-19 at risk, but also those admitted to the hospital for other reasons**.

- *Rationale for the timing of the primary endpoint:* The average time from hospitalization to mechanical ventilatory support is 2-3 days, and average length of hospital stay is 10 days.[32] We considered a 3 day outcome time point to minimize the need for home measurement and early death. However, >24 hours is required to reach steady state levels of drug, and 3 days is likely too early to detect a treatment benefit. We also considered 10 days, but this increases competing risks and decreases the sensitivity and strength of using the continuous primary outcome but increasing the proportion of patients who either die or recover, skewing the data. We

believe 7 days to represent the optimal balance of these competing concerns.

3.4 *Justification of the secondary outcomes:* Patients severely infected with respiratory dysfunction may be at increased risk of volume depletion, acute kidney injury, or the development of hypotension, so we will systematically track these measured instead of relying upon adverse event reporting which introduces the potential for bias. We will measure P/F ratios daily in case our choice of 7 days proves incorrect. SOFA score is a validated measuring of organ failure in critically ill populations that can be determined. Death is clinically important, but we do not believe our sample size is adequately powered to detect a difference in this outcome. Resource utilization and respiratory support are critical in surge capacity situations and of relevance to this disease process. Validated study instruments to assess dyspnea and physical function will assess patient-centered outcomes and strengthen the biologic plausibility of the intervention. The 7-point ordinal scale described is used by other ongoing Covid-19 clinical trials and will allow harmonization of the trial results. Changes in viral load are important to assess the theoretical possibility of losartan induced ACE2 expression leading to increased viral entry, propagation, and shedding.

4.0 Study Interventions / Interventional Agents

4.1 *Intervention arm:* The intervention is losartan, an angiotensin receptor blocker prepared in suspension per the package insert, administered at 50 mg PO twice daily for 10 days (patients with eGFR 30-59 mL/min/1.73 m^2 will be given 50 mg once daily) or until hospital discharge, whichever comes first. The supply of suspension is stable for 4 weeks refrigerated according to the package insert. Any unused doses will be discarded if unused by the 28 day time point.

- *Rationale for the duration of the intervention:* The average duration of mechanical ventilation caused by Covid-19 is approximately 14 days. We expect that if a benefit is observed, it will become apparent in that time frame. We have chosen to stop treatment at the time of discharge as the participant will have already demonstrated significant clinical improvement, and would introduce significant logistical limitations.
- *Rationale for the dose of treatment:* The lowest dose of losartan is 25 mg orally. The maximum clinical dose is 100 mg daily. Losartan has a half-life of 2 hours with an active metabolite E3174 of 6-9 hours. The threshold for blockade of angiotensin-II receptor is at least 20 mg per day.[33] Twice daily dosing has been shown to potentiate angiotensin-II receptor blockade compared with once daily dosing. At this dose, extrapolated data suggests <70% inhibition of angiotensin-II receptors. At the highest maximum allowable dose (50 mg twice daily) we would anticipate >80% inhibition of angiotensin-II receptor. We believe this is necessary as these patients have significant pro inflammatory response. Recent reports indicate that patients with COVID19 have increased plasma Ang II levels and that Ang II levels correlate with COVID19 disease severity and viral load. AT1R inhibition is associated with decreased activity of STAT1 and NFkB downstream signaling as confirmed by western blot analysis. Furthermore, since our initial submission Losartan has been identified for its potential inhibitory effects on SARS-CoV2 viral life cycle based on

molecular docking analysis of the coronavirus macrodomain. We propose a dose increase to 100 mg daily (50 mg PO BID in patients without renal dysfunction). We have previously utilized this dose in previous RCTs in HIV (Schacker/Fletcher). These studies confirmed the safety profile of Losartan with a mean systolic blood pressure (SBP) reduction of only 7 mmHg at 100 mg daily and excellent tolerability and safety profile. Our findings confirm prior reports of 6 mmHg mean SBP reduction for Losartan when doses at 100mg daily. Patients will receive intensive safety monitoring as this will be delivered in the inpatient setting with blood pressure checks multiple times per day and minimum required potassium, creatinine and GFR checks prior to enrollment, day 1, 3, and 7 (or prior to discharge). In addition, patients will have routine creatinine and potassium monitored if ordered clinically We hypothesize significant benefit as 4 preliminary and observational reports out of China and the UK suggest significant reductions in disease severity and inflammatory profile for patients treated with ACEi or ARBS with no preliminary reports suggesting harm from ACEi or ARBS in Covid19 patients.

- We propose a dose of 50 mg twice daily, given significantly improved RAS blockade. This is a higher dose than we are proposing for study in outpatients, as patients will be carefully monitored with regular blood pressures and renal function testing. Furthermore, patients will have a higher severity of illness and are more likely to benefit from more robust blockade. Patients with moderate renal dysfunction (eGFR 30-59 mL/min/1.73 m^2 will be given 50 mg once daily given impaired renal clearance.

4.2 *Placebo arm:* The same volume of suspension prepared according to the package insert will be administered without the active ingredient. Any unused doses of placebo will be discarded if unused by the 28 day time point.

4.3 *Investigational new drug.* The study will be conducted under IND 148365.

4.4 *Timing of the intervention:* Treatment will continue for 10 days or hospital discharge.

4.5 *Duration of study participation:* The final follow-up will occur at 90 days after randomization to determine episodes of delayed hospitalization. Data analysis is expected to be completed within 1 year of enrollment of the final patient.

4.6 *Drug Handling, blinding, and randomization:*
Fairview Investigational Drug Services (IDS), part of M Health Fairview will oversee the preparation of treatment drug and placebo. Identical suspensions (#20) will be prepared in identical syringes and labeled with a deidentified study number coded by site and patient number. A site-specific randomization schema will be created (each site will undergo block randomization in variable permuted blocks by site and age strata). Given significant differences in outcome by age, patients will be randomized by age strata (>=60, <60). Randomization allocation will be 1:1. IDS will not be blinded to patient randomization. All other members of the study, the participants, and the clinical care team will be blinded to treatment allocation.

4.7 *Provision of study drug to patients:* The local Fairview or HCMC pharmacy will dispense the patient specific supply to each patient after consent has been obtained and

a prescription has been received from an authorized prescriber. The investigational drug or placebo will be provided to the bedside nurse twice daily for the duration of study participation.

4.8 *Study unblinding:* MHealth Fairview, East bank Inpatient Pharmacy UMMC campus, will hold the blind for all subjects on study. In the event of an emergency and unblinding is necessary, study staff can contact 612-273-3066, option 1, then option 2. This information is included on each label.

4.9 *Biosafety*: N/A. The intervention is not a biohazard.

4.10 *Stem Cells:* N/A

4.11 *Fetal Tissue:* N/A

4.12 *Use of radiation:* N/A

4.13 *Use of Center for Magnetic Resonance Research:* N/A

5.0 Procedures Involved

5.1 *Screening:* Screening logs containing non-identifiable information (age, gender, race, ethnicity, location of presentation, and reason for exclusion) will be maintained for patients screened but not enrolled. Initial pre-screening to determine study eligibility will necessarily proceed under waiver of informed consent and waiver of HIPAA authorization through the screening of electronic medical records. In addition, sites will have an electronic dashboard of all pending and confirmed positive COVID-19 tests that can be used for real-time prescreening of patients. If the patient appears to be eligible based on pre-screening, and if acceptable to the treating physician, the patient will be approached by a study investigator or delegated study coordinator or research nurse. *For the sake of healthcare provider safety, and to minimize the spread of the virus, this visit will occur via a HIPAA compliant telemedicine consultation or phone.*

5.2 *Enrollment:* Patients or their legally authorized representative, with due consideration of vulnerable patient populations, (see section 9) will be eligible for consent by delegated members of the study team with appropriate training and experience in the conduct of human subjects research. At this visit, consents will be reviewed in detail with the subject or LAR. The subject or LAR will have the opportunity to ask questions. If the patient (or LAR) agrees to become a study participant, electronic consent will be obtained using HIPAA and rule 11 compliant electronic consent, stored and maintained in a secure RedCap server (see departmental policy on eConsent). For patients who are enrolled via a LAR, we will attempt to reconsent the patient for ongoing study participation. If they decline, previous information gathered will be utilized but no further information will be collected. If the patient is unable to be contacted prior to discharge or by phone following discharge, or they do not regain capacity to consent, the participant will continue to participate in study procedures under LAR consent.

- *Rationale for the use of telemedicine / phone consent:* We have chosen this method of consent *in order to minimize risk to research team and healthcare providers, and to decrease community spread of the disease*. We have <u>prior experience</u> using telemedicine and phone consent coupled with electronic consent form review for <u>time-sensitive clinical trials of</u>

stroke. While the *rationale* for eConsent is different in this case (minimization of disease spread during a pandemic), we believe it is *appropriate* for the disease and intervention being studied. Critically, the established safety profile of losartan, with minimal side effects of effects on blood pressure in healthy patients without an overactive renin-angiotension-aldosertone system, combined with the close telemedicine and EMR based follow-up methods proposed in this study make the risk : benefit ratio for the alteration of traditional consent process acceptable for participants, providers, and the public.

5.3 *Administration of study drug:* Study drug will be dispensed to bedside nurses twice daily for administration. Patients unable to take the suspension (due to endotracheal intubation) will have it administered via an orogastric or nasogastric tube, or G-tube. If the patient is unable to receive the study drug for respiratory distress, we will allow for +/- 3 hours from the scheduled time of drug administration. If this is not possible, the patient will not receive that dose, and this will be tracked.

5.4 *Patient follow-up:* Participants will receive remote telemedicine, phone, or in person evaluation by trained study personnel every day for the 10 days of the intervention period to monitor for the development of complications as well as day 15. In addition, electronic medical record labs and notes will be monitoring for the development of AE and SAEs. Data will be documented into a Redcap database. The coordinators will have patients complete 2 surveys at enrollment, day 4, day 10, and day 28 (both attached in supplement), if the patient can cooperate. If not, the reason for failure to complete the survey will be recorded. Surveys conducted within 1 day of the time point will be counted as qualifying and not missing. Patients will be contacted at day 28 (+/3 days) and 90 (+/- 3 days) for intermediate term outcomes. If follow-up procedures are not completed due to inability (patient unavailable or intubated), or due to loss to follow-up, these will not be counted as protocol deviations.

5.5 *Pharmacokinetics:* Patients undergoing this voluntary substudy will have up to another 9 mL of total blood drawn (30 mL total day 1 instead of 21-24 mL day 1. The remainder of the samples will be the same for the duration of the study). Blood will be processed, frozen at -80 C until shipping to Dr. Courtney Fletcher at the University of Nebraska for analysis.

5.6 *Table of events:*

		Study Day																
	*Screening/ Enrollment **	*1*	2	3	4	5	6	7	8	9	10	11	12	13	14	15	28	90
**ELIGIBILITY**																		
Covid-19 Result confirmation	X																	
Inclusion/Exclusion	X																	
Informed Consent	X																	
**PRESCREENING / RANDOMIZATION**																		

Additional labs (pregnancy, Cr, potassium) if applicable	X																	
Randomize subject	X																	
**POST-RANDOMIZATION**																		
Enrollment Day 0 CRFs	X																	
*Dispense / administer study drug***		X	X	X	X	X	X	X	X	X	X							
**MINIMUM STUDY PROCEDURES *(while inpatient, if applicable)#***																		
Daily CRFs		X	X	X	X	X	X	X	X	X	X					X		
Creatinine, Potassium$^{\$}$	X$^{\$}$	X$^{\$}$	X	X$^{\$}$	X	X	X	X$^{\$}$	X	X	X	X	X	X	X	X		
SOFA score assessment	X	X	X	X	X	X	X	X	X	X	X							
Estimated P/F	X	X	X	X	X	X	X	X$^{\&}$	X	X	X					X		
Ordinal scale	X	X	X	X	X	X	X	X	X	X	X					X	X	
Blood draws%		X	X		X		X		X		X					X		
Nasopharyngeal or oropharyngeal swabs%		X			X				X		X					X		
Adverse event assessment		X	X	X	X	X	X	X	X	X	X	X				X		X
PROMIS Survey	X				X						X						X	
Short Form 12 Health Survey	X				X						X						X	
**STUDY PROCEDURES FOLLOWING DISCHARGE *(if applicable)***																		
Assessment of Hospitalization																X	X	
Estimated P/F$^{\&}$								&								X		
Follow-up biospecimens (NP/OP swab + blood draw)																@		
Electronic medical record / death index review																		X
Adverse event assessment																X	X	
PROMIS Survey					X						X						X	
Short Form 12 Health Survey					X						X						X	
**OPTIONAL STUDY PROCEDURES**																		
*Pharmacokinetic Lab Draw****		X																

*Within 48 hours of hospital admission *or* a positive test result, whichever is later,

**In women of child-bearing age, patients must confirm negative test with study personnel prior to beginning treatment as well as confirmation of acceptable birth control method

*** A subset of patients will opt-in to an additional pharmacokinetic substudy

$ Record clinical potassium and creatinine. Required measurements occur pre-enrollment, day 1, day 3 (+/- 1 day), day 7 (+/- 1 day).

Only to be performed if patient is an inpatient. Otherwise move to Study procedures following discharge

% Nasopharngeal swabs and blood draws should be collected if local logistical and biosafety procedures allow. Day 1 and 4 and 10 blood and days 1 and 10 NP or OP swabs are preferred if limited.

@ We will attempt to coordinate follow-up clinic visits within 1-2 weeks from hospital discharge to obtain nasopharygeal and blood biospecimens

& Subject discharged before D7 will be sent home with a pulse oximeter to calculate P/F ratio and a phone call will be made at this time

6.0 Data and Specimen Banking

6.1 *Data management:*

- *Storage and Access:* Data collection is the responsibility of the clinical research staff under the supervision of Drs. Tignanelli and Puskarich. Study data will be recorded within a RedCAP database with study personnel being granted access to the database based on the tasks listed in the Delegation of Authority log. Only deidentified data with no PHI will be exported for analysis. The electronic consent (eConsent) forms, which will be maintained in a University of Minnesota RedCap database with established functionality for this purpose in our group. The database is HIPAA compliant.
- *Monitoring:* A designated study monitor will review 100% of electronic consent documents, study drug administration data, and SAE reporting. Additionally, a random sampling of 5% of subjects will undergo full evaluation and comparison of source documentation with data entered into the study database. The first data monitoring visit will be scheduled once 10% of the cohort has been enrolled, with repeat monitoring after 50% and 100% of enrollments. Monitors will evaluate 100% of users to ensure assigned access is consistent with training and delegation of authority logs. Remote clinical monitoring with source verification of the electronic health record will be allowed given limitations in local site access in the setting of the COVID epidemic limiting in person monitoring consistent with risk-based monitoring principles.
- *Release/Sharing:* Data will be shared among members of the study team with appropriate access assigned in Redcap based on study role, training, and delegation of authority. Data will be made available to assigned study monitors, the data safety monitoring board, the appropriate University of Minnesota oversight authorities, the Food and Drug Administration, and other state and federal regulatory authorities as required by state and federal law.
- *Data sharing:* Requests for data following study completion for sharing will be evaluated by a steering committee that will be established and will

include the principal investigators. Requests will be evaluated on a case-by-case basis. If data are shared, only deidentified data will be shared.

6.2 *Biospecimen management:*

- *Specimen banking:* Biologic specimens (nasopharyngeal or oropharyngeal swabs and blood samples) provided by patients will be transported via courier service with the capacity to transport biologic materials with due consideration of communicable diseases. Specimens will be transported and processed by the University of Minnesota BSL-3 laboratory and frozen at -80 C until the time of analysis. Samples will be deidentified and labeled only with a patient ID number. Other sites will be encouraged to also obtain these specimens using local resources, with transfer of collected materials as they are able.
- *Biospecimens and protocol deviations:* Baseline clinical laboratory results will be required to establish inclusion and exclusion criteria, and missing data will be reported as protocol deviations. However, significant barriers exist to the collection and processing of other biospecimens due to the increased containment required, meaning that not every study site may be able to accomplish these procedures. In addition, patient self-collection will lead to missing samples. While every effort will be made to obtain these biospecimens, failure to do so will not constitute protocol deviations.
- *Specimen sharing:* Requests for specimen sharing during or following study completion for sharing will be evaluated by a steering committee that will be established and will include the principal investigators. Requests will be evaluated on a case-by-case basis. If specimens are shared, human genetic sequencing analysis will only proceed on those patients who opt-in to genetic testing. Genetic testing of viral genomes genomes or epigenetic analysis of human genomes will be allowed to proceed on all patients.

7.0 Sharing of Results with Participants

7.1 *Sharing of results:* Data collected within this study will be obtained from the participants themselves and recorded on collection templates or directly into Redcap, or will be obtained from the electronic medical record and thus are already available to the patient and treatment team. Any testing of biospecimens will occur at a later time point and do have specific clinical relevance and will not be shared with the patient.

7.2 *Sharing of genetic testing*

- *Disclosure of results:* There is no plan to disclose genetic results to the patients as none is yet planned.
- *If returning results to participants:* N/A

7.3 *Future analysis of genotypes:* Genetic sequencing testing, while potentially an area of future investigation as a means to determine if participants with specific genetic differences are more or less susceptible to COVID-19, is not currently planned nor funded. As such, data will not be disclosed to the participants. To the extent possible in

future work, participants or families with rare variants will undergo attempts at anonymization.

8.0 Study Population

8.1 *Inclusion Criteria*

- Presumptive positive laboratory test for Covid-19 based on local laboratory standard
- Age greater than or equal to 18 years of age
- At least one symptom of coronavirus as utilized by the CDC within 24 hours of enrollment (https://www.cdc.gov/coronavirus/2019-ncov/symptoms-testing/symptoms.html). [Currently those symptoms include: Fever or chills, cough, shortness of breath or difficulty breathing, fatigue, muscle or body aches, headache, new loss of taste or smell, sore throat, congestion or running nose, nausea or vomiting, or diarrhea]
- Admission to the hospital with a respiratory SOFA >=1 *and* increased oxygen requirement compared to baseline among those on home O2
- Randomization within 48 hours of presentation of hospital admission *or* within 48 hours of a positive test result, whichever is later

8.2 *Rationale for inclusion criteria:* This is a pragmatic trial looking to enroll a diverse cohort of participants so the results may be broadly applicable to a wide patient population if efficacious. We do not have sufficient data to support lack of efficacy in a specific subgroup at this time to justify their exclusion. However, a respiratory SOFA of at least 1 is included to provide room for improvement to meet the primary efficacy endpoint. Patients on home oxygen therapy must demonstrate an acute deterioration under the same rationale. While we currently propose to exclude children due to insufficient time at the writing of this protocol to closely scrutinize safety and dosing in that group, along with logistical challenges of having different doses, we have plans to evaluate the possibility of expanding to include that patient population as soon as logistically feasible.

8.3 *Exclusion Criteria*

- Randomization > 48 hours of admission order or positive test result, whichever is later
- Currently taking an angiotensin converting enzyme inhibitor (ACEi) or Angiotensin receptor blocker (ARB)
- Prior reaction or intolerance to an ARB or ACE inhibitor, including but not limited to angioedema
- Pregnant or breastfeeding women
- Lack of negative urine or serum pregnancy test
- Not currently taking a protocol allowed version of contraception: intrauterine device, Depo-formulation of hormonal contraception (e.g. medroxyprogesterone acetate / Depo-Provera), subcutaneous contraceptive (e.g. Nexplanon), daily oral contraceptives with verbalized commitment to taking daily throughout the study period; use of condoms

or agree to abstain from sexual intercourse during the study. All women of child bearing age enrolled in this fashion will be informed of the teratogenic risks. If enrolled under LAR, they will be informed of the risks after regaining capacity.

- Patient reported history or electronic medical record history of kidney disease, defined as:
    - Any history of dialysis
    - History of chronic kidney disease stage IV
    - Estimated Glomerular Filtration Rate (eGFR) of < 30ml/min/1.73 m^2 at the time of randomization
- Severe dehydration at the time of enrollment in the opinion of the investigator or bedside clinician
- Most recent mean arterial blood pressure prior to enrollment <65 mmHg
- Patient reported history or electronic medical record history of severe liver disease, defined as:
    - Cirrhosis
    - History of hepatitis B or C
    - Documented AST or ALT > 3 times the upper limit of normal measured within 24 hours prior to randomization
- Potassium >5.0 within 24 hours prior to randomization *unless* a repeat value was <=5.0
- Treatment with aliskiren
- Inability to obtain informed consent from participant or legally authorized representative
- Enrollment in another blinded randomized clinical trial for COVID

8.4 *Rationale for exclusion criteria:* We expect treatment results to be available within 24 hours of testing given the acuity of the patient, though delays are expected so this window is open until 48 hours for logistical considerations. We wish to limit interventions to an early window, as progression from admission to mechanical ventilatory support occurs within days, and we intend to maximize potential efficacy of the treatment to mitigate respiratory failure. Patients already taking or with prior adverse events to either an ACE inhibitor or ARB are excluded for risk of side effects. Losartan is a class D drug. Women of child bearing age (14-60) without a history of hysterectomy must have a documented negative pregnancy test in the emergency department *or* have had a prior history of hysterectomy. We will exclude patients with significantly elevated creatinine or liver enzymes expected to affect drug clearance. Aliskiren is contraindicated for losartan co-treatment. Patients with hypotension will be excluded given an expected decrease of 6-8 mmHg in their blood pressure that might necessitate the need for cardiovascular interventions. Finally, all patients or their legally authorized representative will give written informed consent.

9.0 Vulnerable Populations

9.1 *Vulnerable Populations:* By nature of the disease and the fact COVID-19 is a rapidly evolving pandemic, with uncertain clinical course but potentially significant morbidity and mortality, all participants enrolled in this study are considered to be vulnerable. Some participants have additional characteristics that add to this vulnerability. Our criteria for inclusion and exclusion of each of these groups is summarized below, followed by our rationale and additional safeguards built into our study design. Our group has significant experience enrolling vulnerable patients in emergency care trials, and experience with a diverse population of potential participants in high stress situations.

Population / Group	Identify whether any of the following populations will be targeted, included (not necessarily targeted) or excluded from participation in the study.
Children	Excluded from Participation
Pregnant women/fetuses/neonates	Excluded from Participation
Prisoners	Excluded from Participation
Adults lacking capacity to consent and/or adults with diminished capacity to consent, including, but not limited to, those with acute medical conditions, psychiatric disorders, neurologic disorders, developmental disorders, and behavioral disorders	Included/Allowed to Participate
Non-English speakers	Included/Allowed to Participate
Those unable to read (illiterate)	Included/Allowed to Participate
Employees of the researcher	Included/Allowed to Participate
Students of the researcher	Included/Allowed to Participate
Undervalued or disenfranchised social group	Included/Allowed to Participate
Active members of the military (service members), DoD personnel (including civilian employees)	Included/Allowed to Participate
Individual or group that is approached for participation in research during a stressful situation such as emergency room setting, childbirth (labor), etc.	Included/Allowed to Participate
Individual or group that is disadvantaged in the distribution of social goods and services such as income, housing, or healthcare.	Included/Allowed to Participate

Individual or group with a serious health condition for which there are no satisfactory standard treatments.	Included/Allowed to Participate
Individual or group with a fear of negative consequences for not participating in the research (e.g. institutionalization, deportation, disclosure of stigmatizing behavior).	Included/Allowed to Participate
Any other circumstance/dynamic that could increase vulnerability to coercion or exploitation that might influence consent to research or decision to continue in research.	Excluded from Participation

9.2 *Additional safeguards:*

- *Non-English speakers:* Excluding this group would compromise the principle of justice in clinical research. For groups likely to be enrolled given our known demographics (Spanish-speaking and Somali-speaking), we will translate informed consent forms (ICF) and use professional interpreters during both the consent and follow-up periods. These interpreters are available to our group during daytime hours by telephone. *We will request the use of the Short Form Consent for non-English speaking particiapnts, and adhere to the regulations surrounding its use. When the translated ICsF become available, they will be used for non-English speakers.*
- *Employees or students of the researcher:* To minimize the potential for coercion, any employee or student of the research will undergo screening and informed consent by study personnel, but not by the investigator who is the direct supervisor of the vulnerable participant. The screening will not be performed by the study physicians or investigators, so this group may opt out in a deidentified fashion at that stage. Furthermore, if the potential participant wants to enroll, all interactions will occur with a different study investigator (such as the other co-principal investigator. These two clinical investigators work in different departments with different staff).
- *Undervalued or disenfranchised social group:* We will assure confidentiality, that they have the right to withdraw at any time without penalty, and they have the freedom to refuse to answer questions.
- *Active members of the military (service members), DoD personnel (including civilian employees):* The status of participants of this group is not asked during the study nor known by study staff, and therefore vulnerability is not increased for anyone in this group.
- *Individual or group that is disadvantaged in the distribution of social goods and services such as income, housing, or healthcare:* We will assure participants that participation in this study will be treated with the strictest confidentiality, that they have the right to withdraw at any time without penalty, and they have the freedom to refuse to answer questions.
- *Individual or group with a serious health condition for which there are no satisfactory standard treatments:* We will assure that information from participants in this study will be treated with the strictest confidentiality, that

participants have the right to withdraw at any time without penalty, and they have the freedom to refuse to answer questions.

- *Individual or group with a fear of negative consequences for not participating in the research (e.g. institutionalization, deportation, disclosure of stigmatizing behavior):* The status of participants from these groups is not asked during the study nor known by study staff, and that vulnerability is not increased for anyone in this group.

10.0 Local Number of participants

10.1 *Locations:* Enrollment will occur on the ED (or designated COVID19 areas) of participating study sites see section 22.1).

10.2 *Number of participants:* We expect to enroll a minimum of 200 participants, with up to 220 participants when considering screen failures and withdrawals.

11.0 Local Recruitment Methods

11.1 *Recruitment Process:* See section 5.0

11.2 *Identification of Potential Participants:* See section 5.0

11.3 *Recruitment materials:* N/A

1.5 *Payment:* Patients will be eligible for a $25 gift card for their time to return for the 15-day blood draw for renal function testing if allowed by local site.

12.0 Withdrawal of Participants

12.1 *Withdrawal Circumstances:* Participants may be withdrawn from research without their consent if they develop SAEs that the investigator believes to be likely related to the study drug. Participants may also withdraw from the study at any time for any reason. All patients withdrawn from the study will be analyzed using intent to treat principles.

12.2 *Withdrawal Procedures:* If patients choose to withdraw, study procedures including intervention and follow-ups will stop. Given the risks of transmission of infectious disease, written notification is not required. If the patient chooses to withdraw, the study team will ask if the patient is willing to allow continued electronic medical record evaluation for the determination of study endpoints (partial withdrawal). We will also ask if the patient wishes to withdraw consent for the use of future biologic specimens. If the patient declines to participate in any component of research, no further study data will be collected, biologic samples will be destroyed, but existing study data may be used. Each of these will be documented and stored as a study document.

12.3 *Termination Procedures:* Upon trial termination, study outcomes will be followed until completion. Case report forms and collection templates will be maintained in paper format (when application) and electronically for 7 years in accordance with regulations. Collected data will be used for secondary analyses with approval of the principal investigators in a deidentified fashion. Data entry will be completed, checked for accuracy, and locked for analysis within 1 year of enrollment of the final patient.

13.0 Risks to Participants

13.1 *Foreseeable Risks:* Potential risks of losartan are detailed in consent forms. The most severe risks of losartan use include teratogenicity in the setting of pregnancy, hypersensitivity including angioedema, symptomatic hypotension, worsening of renal function, and electrolyte abnormalities. The most common side effects of losartan include fatigue, weakness, diarrhea, chest pain and anemia. Subjects will be rigorously screened to ensure that they are at low risk for complications due to losartan and they will be monitored closely for side effects and signs of toxicity. The package insert for Losartan has been included with this protocol. Clinical trial and post-marketing surveillance data (see package insert) demonstrate an excellent safety profile. In over 4,000 patients, there was a low incidence of adverse events (2.3%) comparable to placebo (3.7%). The effects more common in losartan than placebo were clinically minor, including dizziness (3% vs 2%), upper respiratory infection (8% vs 7%), nasal congestion (2% vs 1%) and back pain (2% vs 1%). While losartan has the potential to cause symptomatic hypotension, four studies have identified losartan has a negligible effect on resting blood pressure and heart rate in healthy volunteers.[33-36] While there may be a higher risk in critically ill patients, we will exclude patients with bedside clinician assessment of volume depletion that might increase the risk of side effects. It has been theorized there may be an increase risk of cardiac dysfunction in patients taking an ACE-I or ARB in COVID-19, though evidence for this effect is lacking. We will monitor troponin and BNP/pro-BNP levels in participants and report these data to the DSMB if measured clinically. In fact, the most recent preprinted (non-peer reviewed) data from China now suggest a protective effect based on retrospective data compared to other antihypertensive regiments. Nevertheless, based on this recent suggestion, we will track troponin and BNP/proBNP measurements in addition to our previously planned assessments of new episodes of hypotension and shock, and report these data to the DSMB. In addition, all patients are closely monitored for clinical purposes given their severity of illness, combined with daily assessments for AEs and SAEs will allow for early detection of side effects and reduce the risks to patients.

13.2 *Reproduction Risks:* Losartan is class D in pregnancy. Women of childbearing age will only be eligible for enrollment with a negative pregnancy test prior to enrollment. In addition, women breastfeeding will be excluded from the study.

13.3 *Risks to Others:* The primary risk to others is the risk to study personnel of becoming infected with COVID-19. To overcome this barrier, patients will be enrolled using an electronic consent process already established among our group for other studies. In addition, follow-up evaluations will preferentially be performed by telemedince evaluation or phone evaluation. If a face-to-face evaluation or physical examination is required due to concern for adverse events, investigators trained and fitted with N95 masks or equivalent will perform a face-to-face examination. Participants will generate potential biohazards (oropharyngeal swabs), the safety steps surrounding which are discussed in section 6.2.

14.0 Potential Benefits to Participants

14.1 *Potential Benefits:* If randomized to the intervention arm, and if losartan is demonstrated to prevent the progression of acute lung injury, patients may benefit by having reduced lung injury, decreased need for oxygen or mechanical ventilation, or possibly lower mortality. If randomized to the control arm, there is no

direct potential benefit to the patient. However, the proposed research has the potential to significantly benefit people infected with COVID-19.

15.0 Statistical Considerations

15.1 *Data Analysis Plan:* All analysis will focus on comparisons between the treatment and control groups. Demographics and other baseline clinical characteristics will be summarized using descriptive statistics. Descriptive statistics will be presented by treatment group using mean, standard deviation, median, and interquartile range (IQR) for continuous variables and frequencies and percentages for categorical variables. The primary outcome, change in estimated P/F ratio at 7 days, will be summarized by group using the sample mean with 95% asymptotic confidence intervals. Patients discharged home will be given a pulse oximeter and called for the value at 7 days, adjusted for home O2 use as applicable. Patients who died will be penalized with a P/F of 0 (lower values indicate higher degree of respiratory failure) in order to address competing risks. Like respiratory SOFA, an adjustment is applied based on the use of PEEP. For the primary statistical analysis, Treatment groups will be compared using the two-sample t-test for unequal variances and the treatment effect will be summarized by the mean difference between groups. As a secondary statistical approach, we will also jointly model the longitudinal and time to event outcome under the assumption of informative missingness, jointly modeling P/F ratio measured daily and time to death using a shared random effects model. Finally, we will analyze these data on an ordinal scale (the respiratory SOFA score), which is a 0-4 ordinal scale derived from the estimated P/F ratio. In addition, patients who die prior to the day 7 assessment will be assigned a 5 for this analysis.

Secondary endpoints will be summarized by the sample mean or sample proportion, as appropriate, and compared using the unequal variance two-sample t-test or Chi-square test, as appropriate. Appropriate data transformations will be used to assure that assumptions of all statistical tests are met. Time-to-event endpoints will be summarized by Kaplan-Meier curves and compared using Cox proportional hazards regression. For all endpoints, we will complete secondary analyses comparing for important baseline variables that are unequally distributed between treatment groups. Preplanned subgroup analyses are planned by age (dichotomized at 50 and by decade), history of hypertension, sex, and smoking history, timing of randomization from symptom onset, and respiratory support needed at the time of randomization.

15.2 *Power Analysis:* Assuming a fixed sample size, we analyzed power using a number of assumptions. In general, studies with a similar degree of acute lung injury that we expect in this study have a standard deviation of estimated P/F ratio of 100-125. ARDS trials tend to have smaller SD due to narrowed inclusion criteria, while a single study of H1N1 with a lower severity of illness had a SD of 150. We calculated power for a variety of assumptions. If the SD is 125 or less, we will have >0.8 power to detect a difference in P/F of 50. Differences of less than this are of uncertain clinical significance. This method and outcome has more power than our alternative approach of respiratory SOFA, which is a more crude measure of the estimated P/F ratio.

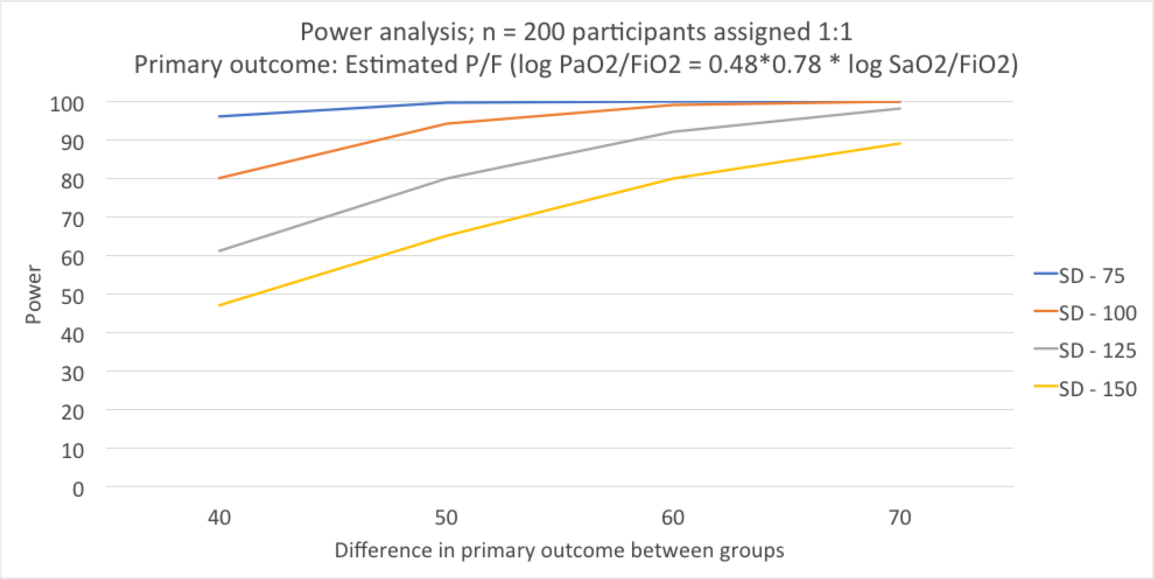

15.3 *Data Integrity:* The principal investigator will manage oversight of quality control for collected data. Recruitment of subjects will be performed using inclusion/exclusion criteria defined above. Study data will be recorded within a RedCAP database, which is HIPAA compliant, password protected with variable assignable security access and an audit trail consistent with all regulatory requirements for maintenance of clinical trial data. Data summaries and quality control checks will be run routinely.

16.0 Health Information and Privacy Compliance

16.1 **Select which of the following is applicable to your research:**

☐ My research does not require access to individual health information and therefore assert HIPAA does not apply.

☒ I am requesting that all research participants sign a HIPCO approved HIPAA Disclosure Authorization to participate in the research (either the standalone form or the combined consent and HIPAA Authorization).

☐ I am requesting the IRB to approve a Waiver or an alteration of research participant authorization to participate in the research.

☒ An external IRB (e.g. Advarra) is reviewing and we are requesting use of the authorization language embedded in the template consent form in lieu of the U of M stand-alone HIPAA Authorization. Note: External IRB must be serving as the privacy board for this option.

16.2 **Identify the source of Private Health Information you will be using for your research (Check all that apply)**

☐ I will use the Informatics Consulting Services (ICS) available through CTSI (also referred to as the University's Information Exchange (IE) or data shelter) to pull records for me

☒ I will collect information directly from research participants.

☐ I will use University services to access and retrieve records from the Bone Marrow Transplant (BMPT) database, also known as the HSCT (Hematopoietic Stem Cell Transplant) database.

☒ I will pull records directly from EPIC.

☐ I will retrieve record directly from axiUm / MiPACS

☐ I will receive data from the Center for Medicare/Medicaid Services

☒ I will receive a limited data set from another institution

☒ Other. Describe: CTSI's BPIC will be used to pull structured data directly from EPIC into redcap (ED vitals, labs, etc.), or data will be transcribed by coordinators into our Redcap research database. Study coordinators will contact patients daily to obtain data and will input it directly into the Redcap research database.

16.3 *Explain how you will ensure that only records of patients who have agreed to have their information used for research will be reviewed:* Study coordinators and BPIC will review if patients have opted out of research by using the opt-out flag within Epic to determine research status. Only patients that have opted-in to research or have expressed that they wish to delay opt-out status will be included in this study.

16.4 *Approximate number of records required for review:* 220.

16.5 *Please describe how you will communicate with research participants during the course of this research. Check all applicable boxes:*

☐ This research involves record review only. There will be no communication with research participants.

☒ Communication with research participants will take place in the course of treatment, through MyChart, or other similar forms of communication used with patients receiving treatment.

☐ Communication with research participants will take place outside of treatment settings. If this box is selected, please describe the type of communication and how it will be received by participants.

16.6 *Explain how the research team has legitimate access to patients/potential participants:* Informed consent will be obtained from patients granting access to clinical data for study participants.

16.7 *Location(s) of storage, sharing and analysis of research data, including any links to research data (check all that apply).*

☒ In the data shelter of the Information Exchange (IE)

☒ Store ☒ Analyze ☒ Share

☐ In the Bone Marrow Transplant (BMT) database, also known as the HSCT (Hematopoietic Stem Cell Transplant) Database

☐ Store ☐ Analyze ☐ Share

☒ In REDCap (recap.ahc.umn.edu)

☒ Store ☒ Analyze ☐ Share

☐ In Qualtrics (qualtrics.umn.edu)

☐ Store ☐ Analyze ☐ Share

☐ In OnCore (oncore.umn.edu)

☐ Store ☐ Analyze ☐ Share

☐ In the University's Box Secure Storage (box.umn.edu)

☐ Store ☐ Analyze ☒ Share

☐ In an AHC-IS supported server. Provide folder path, location of server and IT Support Contact:

☐ Store ☐ Analyze ☐ Share

☐ In an AHC-IS supported desktop or laptop.

Provide UMN device numbers of all devices:

☐ Store ☐ Analyze ☐ Share

☐ Other. Describe:

Indicate if data will be collected, downloaded, accessed, shared or stored using a server, desktop, laptop, external drive or mobile device (including a tablet computer such as an iPad or a smartdevice (iPhone or Android devices) that you have not already identified in the preceding questions

☐I will use a server not previously listed to collect/download research data

☐I will use a desktop or laptop not previously listed

☐I will use an external hard drive or USB drive ("flash" or "thumb" drives) not previously listed

☐I will use a mobile device such as an tablet or smartphone not previously listed

16.8 *Consultants. Vendors. Third Parties.* N/A

16.9 *Links to identifiable data:* All information that identifies study subjects will be handled in accordance with regulatory bodies including HIPAA regulations and the cIRB. This information will be made only available to the principal investigators and study personnel who directly participate in the research calls. Prior to enrollment, participants will sign an authorization to use and disclose PHI for research purposes. All staff will have been trained in the use of PHI. Subjects will be assigned a random subject ID number and a link between identifiers will be maintained in an excel log which will be stored in a secure folder in the AHC-IE data shelter, the University of Minnesota's most secure healthcare research

environment. Only the principal investigators and study nurses will have access to this file.

16.10 *Sharing of Data with Research Team Members:* A fully de-identified (of all PHI) database will be generated which will be used for statistical analysis and for monitoring by our DSMB. This database will exist within Redcap.

16.11 *Storage and Disposal of Paper Documents:* At this time we do not anticipate any paper documents related to this study. Everything, including consents will be done electronically. If the need for paper collection templates becomes evident, these documents will be stored in locked rooms where only study personnel have access. They will be maintained for 7 years as legally required, after which time they will either be destroyed or moved to long-term storage.

17.0 Confidentiality

17.1 *Data Security:* All data will be stored on servers (Redcap and CTSI AHC-IE data shelter) managed by the University of Minnesota IS. No data will be stored on individual research computers, flash drives, etc. These servers Redcap and the data shelter meet all data regulatory requirements and are HIPAA compliant.

18.0 Provisions to Monitor the Data to Ensure the Safety of Participants

18.1 *Integrity Monitoring:* Data entry will be performed by trained members of the research team who have received documented delegation of authority. Data integrity will be overseen by the PIs and the Minnesota CCBR. A study monitor will review 100% of electronic consent documents, study drug administration data, and SAE reporting. Additionally, a random sampling of 5% of subjects will undergo full evaluation and comparison of source documentation with data entered into the study database. The first data monitoring visit will be scheduled once 10% of the cohort has been enrolled, with repeat monitoring after 50% and 100% of enrollments. Monitors will evaluate 100% of users to ensure assigned access is consistent with training and delegation of authority logs. The project research coordinators at the sites will maintain binders containing pertinent documents and information including IRB approval letters, documentation of GCP training, licenses, human subjects research ethics training certificates, curriculum vitae, and correspondence.

18.2 *Adverse events reporting*

- *Definition of an Adverse Event (AE):* An AE is any symptom, sign, illness, or experience that develops or worsens in severity during the course of the study but does not necessarily have a causal relationship with study agent/intervention or procedures. A clinical trial adverse event is defined as any untoward medical event associated with the use of a drug or study procedure. The Investigators will determine daily if any adverse events occur during the period from enrollment (signing of the informed consent) through study day 15. Investigators will determine if the event is serious or related to the study drug. The rationale for this time window is the half-life of 2 hours for losartan and 6-9 hours for its metabolite. Even in the setting of impaired clearance, 3 days after study drug cessation is more than adequate to help to define the period at risk from losartan. Abnormal

results of diagnostic procedures are considered to be AEs if the abnormality:

- results in study withdrawal,
- is associated with a serious adverse event (SAE),
- leads to additional treatment or to further diagnostic tests, or
- is considered by the Investigator to be of clinical significance.

- *Definition of a Serious Adverse Event*: Adverse events are classified as serious or non-serious. An SAE is any AE that is:
  - *Fatal,*
  - *life-threatening,*
  - *requires or prolongs a hospital stay,*
  - *results in persistent or significant disability or incapacity,*
  - *a congenital anomaly or birth defect, or*
  - *an important medical event.*

    Important medical events are those that may not be immediately life threatening, but are clearly of major clinical significance. They may jeopardize the subject, and may require intervention to prevent 1 of the other serious outcomes noted above. For example, drug overdose or abuse, a seizure that did not result in in-patient hospitalization, or intensive treatment of bronchospasm in an emergency department would typically be considered serious. Serious adverse events will be collected during the first 15 study days, regardless of the investigator's opinion of causation. Thereafter, serious adverse events are not required to be reported unless the investigator feels the events were related to either study drug or a protocol procedure.

  Study site personnel must alert a study investigator of any serious and study drug or study procedure related adverse event within 24 hours of investigator awareness of the event. Alerts issued via telephone are to be immediately followed with official notification on the adverse event case report form.

  All AEs that do not meet any of the criteria for serious should be regarded as non-serious AEs.

- *Preexisting Condition:* A preexisting condition is one that is present at the start of the study. A preexisting condition will be recorded as an AE if the frequency, intensity, or the character of the condition worsens during the study period.
- *Post-study Adverse Event:* All unresolved AEs will be followed by the Investigator until the events are resolved, the subject is lost to follow-up, or the AE is otherwise explained. At the last scheduled visit, study personnel will instruct each subject to report any subsequent event(s) within 3 months of study completion that the subject, or the subject’s

personal physician, believes might reasonably be related to participation in this study.

- *Abnormal Laboratory Values:* A clinical laboratory abnormality should be documented as an AE if any 1 of the following conditions is met:
  - The abnormality suggests a disease and/or organ toxicity that worsened from the time of enrollment, or
  - The abnormality is of a degree that requires active management; e.g., change of dose, discontinuation of the drug, more frequent follow-up assessments, further diagnostic investigation, etc.
- *Continuous SAEs of interest monitoring:* The following SAEs will be continuously reported to the DSMB: deaths. Cardiovascular events (need for vasoactive drugs or fluids for hypotension), and respiratory events (worsening hypoxia, worsening acute respiratory distress syndrome, or new respiratory failure) will be continuously reported to the DSMB within 24 hours of recognition for safety reasons. While they will be reported continuously, they will not necessarily be filed as SAEs unless the investigator deems it to be related to study procudures (see below)
- *Other outcomes that may be exempt from adverse event reporting:* Study specific outcomes as outlined in 5.1 and 5.2 are exempt from adverse event reporting unless the investigator deems the event to be related to the study procedures (or of uncertain relationship) or if the event leads to discontinuation of study procedures. The following are examples of events that will be considered study specific clinical outcomes and will be systematically tracked through the study design. The rationale for this criteria is that these events are likely to be present in a large proportion of the study population, and relatedness will be subject to significant subjective assessment, making adverse event reporting can be sporadic and inconsistent. Therefore, events more likely to be truly related to a study intervention are better treated by consistent tracking through the systemic design and collection of potentially related adverse events:
  - Respiratory events: decreased PaO2/FiO2, hypoxia, worsening acute respiratory distress syndrome, or respiratory failure.
  - Cardiovascular events: (need for vasoactive drugs or fluids for hypotension)
  - Hepatic events: hepatic injury or liver dysfunction that leads to an increase from baseline in the serum level of bilirubin.
  - Renal events: renal failure, renal insufficiency, or renal injury that leads to an increase from baseline in serum creatinine.
  - Hematologic/coagulation events: coagulopathy, disseminated intravascular coagulation, thrombocytopenia, or thrombocytosis.
  - Changes in serum sodium and potassium
- *Scales Used to Grade Severity of Adverse Events:* All AEs will be graded in the following manner:
  - Grade 1 (Mild): Events require minimal or no treatment and do not interfere with the participant’s daily activities.
  - Grade 2 (Moderate): Events result in a low level of inconvenience or concern. Moderate events may cause some interference with functioning.

- Grade 3 (Severe): Events interrupt a participant's usual daily activity and may require systemic drug therapy or other treatment. Severe events are usually incapacitating.
- Grade 4 (Life-threatening): Any adverse drug experience that places the participant, in the view of the Investigator, at immediate risk of death from the reaction as it occurred (i.e., it does not include a reaction that had it occurred in a more severe form, might have caused death).
- Grade 5 (Death)

- *Scales Used Attribute Adverse Events*: The Principal Investigator will assess the relationship of all AEs to any drug or study procedure:
  - <u>Definitely Related</u>: There is clear evidence to suggest a causal relationship, and other possible contributing factors can be ruled out. The clinical event, including an abnormal laboratory test result, occurs in a plausible time relationship to study agent/intervention administration and cannot be explained by concurrent disease or other drugs or chemicals. The response to withdrawal of the study agent/intervention (de-challenge) should be clinically plausible. The event must be pharmacologically or phenomenologically definitive, with use of a satisfactory re-challenge procedure if necessary.
  - <u>Probably Related</u>: There is evidence to suggest a causal relationship, and the influence of other factors is unlikely. The clinical event, including an abnormal laboratory test result, occurs within a reasonable time sequence to administration of the study agent/intervention, is unlikely to be attributed to concurrent disease or other drugs or chemicals, and follows a clinically reasonable response on withdrawal (de-challenge). Re-challenge information is not required to fulfill this definition.
  - <u>Possibly Related</u>: There is some evidence to suggest a causal relationship (e.g., the event occurred within a reasonable time after administration of the trial medication). However, the influence of other factors may have contributed to the event (e.g., the subject's clinical condition, other concomitant events). Although an adverse drug event may rate only as "possible" soon after discovery, it can be flagged as requiring more information and later be upgraded to probable or certain as appropriate.
  - <u>Unlikely:</u> A clinical event, including an abnormal laboratory test result, whose temporal relationship to study agent/intervention administration makes a causal relationship improbable (e.g., the event did not occur within a reasonable time after administration of the trial medication) and in which other drugs or chemicals or underlying disease provides plausible explanations (e.g., the subject's clinical condition, other concomitant treatments).
  - <u>Not related</u>: The AE is completely independent of study agent/intervention administration, and/or evidence exists that the event is definitely related to another etiology. There must be an alternative, definitive etiology documented by the clinician.

- *Response to possibly, probably, or definitely related AEs and SAEs:* SAEs determined to be probably or definitely related will prompt discontinuation of study drug. AEs determined to be probably or definitely related with prompt a) decrease in study drug from twice a day to once a day if currently dosing twice daily, b) discontinuation of study drug if only taking once daily, or c) discontinuation for study drug at the discretion of the investigator.
- *Anticipated Adverse Events*: Adverse events are not expected from any of the study procedures. We do not expect any serious adverse events due to losartan.
- *Recording and Documentation*
  - At each contact with the subject, personnel must seek information as to discomforts or adverse experiences by specific questioning and, as appropriate, by examination. This will typically occur via phone or telemedicine due to concerns regarding infectious contagion. Information about AEs to be recorded includes event description, time of onset, investigator assessment of severity, relationship to Study Agent(s)/Intervention(s), and time of resolution/stabilization of the event. All AEs occurring during the study period (from time of consent signing to study day 15) must be documented appropriately regardless of relationship to study products or procedures unless included on the list of clinical outcomes that may be exempt from adverse outcomes reporting as part of the study protocol and outcomes. Information on all identified AEs will be recorded within 5 days of recognition in the appropriate AE module of the case report form (CRF) on Redcap, and within 24 hours in the event of a SAE.

18.3 *Data Safety Monitoring:* A data safety monitoring board (DSMB) has been convened and includes 4 individuals meeting the key element criteria below, and includes a biostatistician with prior clinical trials DSMB experience. The DSMB will follow the guidance as outlined in the application and the attached charter unless there is a compelling, unanticipated reason to deviate from these rules. Data regarding the primary outcome (hospitalization) will be reported to the DSMB continuously, consistent with continuous study monitoring principles. The DSMB may recommend stopping the trial early only for safety, following these guidelines:

[Patient Safety] A recommendation to suspend, alter the study, or stop for harm would occur if during continuous monitoring there is strong evidence that the rate of IRB-reported SAEs was significantly higher in the experimental group than in the placebo group. SAEs (including increasingly level of ventilator support and initiation of vasopressors, the two SAEs of interest) will be reported within 24 hours, allowing for continuous study monitoring. For the inpatient cohort, we propose formal interim analyses for safety at 10%, 20%, and 30% of enrollment. A 50% preplanned interim analysis for safety, efficacy, and futility is planned with specific stopping rules provided to the DSMB as a guide. Assuming a baseline mortality rate of 10%, this corresponds to formal hypothesis testing after every ~2 events initially, and decreasing as data accumulate. If the observed number of events are significantly lower than expected, the DSMB retains authority to decrease the frequency of monitoring (in terms of number of patients enrolled) to

maintain safety monitoring every ~5 events initially, later expanding to every 10 events. Weekly summary emails of updated AE/SAE rates are provided to the DSMB. Despite the lower number of formal preplanned analyses, the DSMB will still receive near *real-time reporting* of *deaths*, and can deviate from this strategy and perform earlier testing if desired. They retain authority to change this plan if the continuous reporting suggests harm prior to the first formal planned assessment if they feel that a formal hypothesis test is warranted. In addition, the DSMB may request additional safety assessments after the 50% assessment, if they feel that additional formal testing is needed.

[Efficacy] – A preplanned interim analysis at 50% of enrollments will assess for early efficacy or futility.

The DSMB will operate in accordance with guidelines established by the FDA in "Guidance for Clinical Trial Sponsors: Establishment and Operation of Clinical Trial Data Monitoring Committee" jointly published by the CBER, CDER and CDRH of the FDA (https://www.fda.gov/regulatory-information/search-fda-guidance-documents/establishment-and-operation-clinical-trial-data-monitoring-committees) . The key elements include:

1. Expertise and independence: The members must have expertise in the area of study, not be study authors and have no financial conflict of interest.

2. Members will sign a confidentiality agreement.

3. It is preferred that the DSMB have a face-to-face initial meeting, but can meet by telephone thereafter more frequently.

4. All meetings must follow standard operating procedures (SOPs).

If the DSMB recommends closure of the study, then the DSMB Chair will email the principal investigator and IRB. If the trial is stopped for patient safety and a repairable cause of unacceptably high adverse events can be identified, the action must be reviewed and approved by the DSMB and IRB prior to resuming enrollment. For example, if one additional exclusion criterion is discovered that would account for the majority of unexpected events, this new exclusion criterion could be added, and the study could proceed.

19.0 Provisions to Protect the Privacy Interests of Participants

19.1 *Protecting Privacy:* Conversations will be held in a private room whenever possible. Telephone calls will be performed one on one. The electronic consent process takes place using a secure RedCAP server that is HIPAA compliant.

19.2 *Access to Participants:* Conduct of the study and determination of both safety and efficacy of the intervention requires access of the medical records. All study personnel undergo human subjects protection training and understand and value privacy standards set forth by HIPAA. Participants enrolled in the trial are made aware during the informed consent procedure that study personnel will be required to access this private information for trial conduct, and consent to this access prior to agreeing to participate.

20.0 Compensation for Research-Related Injury

20.1 *Compensation for Research-Related Injury:* There is no compensation available for research related injury from the study team. Due to the coronavirus public health

crisis, the federal government has issued an order that may limit your ability to recover damages if you are injured or harmed while participating in this COVID-19 clinical study. If the order applies, it limits your ability to recover damages from the University, researchers, healthcare providers, study sponsor, manufacturer or distributor involved with the study. However, the federal government has a program that may provide compensation to you or your family if you experience serious physical injuries or death due to this study. To find out more about this "Countermeasures Injury Compensation Program" go to https://www.hrsa.gov/cicp/about/index.html or call 1-855-266-2427.

20.2 *Contract Language:* N/A.

21.0 Consent Process

21.1 *Consent Process (when consent will be obtained):* All subjects participating in this study or their LARs will be required to provide prospective informed consent using an eConsent platofrm. Prior to doing so, will discuss the study with study personnel who have been specially trained in obtaining informed consent. Our team has significant experience in this process, with due consideration of vulnerable patient populations. The subject or LAR will review the eConsent in detail and have the chance to ask questions. The personnel will then ask the subject questions to ensure that they understand the information presented in the consent discussion using a teach back method. The rights and welfare of the participants will be protected by emphasizing to them that the quality of their medical care will not be adversely affected if they decline to participate in this study. A copy of the informed consent document will be available electronically to the participants for their records. The participants may withdraw consent at any time throughout the course of the trial (see withdrawal procedures). The eConsent forms are included in this IRB submission.

21.2 *Waiver or Alteration of Consent Process (when consent will not be obtained):* N/A

21.3 *Waiver of Written/Signed Documentation of Consent (when written/signed consent will not be obtained):* N/A

21.4 *Non-English Speaking Participants:* Spanish and Somali speaking persons, who represent the vast majority of non-English speaking patients in the study hospitals and clinics, will be included as described above.

21.5 *Participants Who Are Not Yet Adults (infants, children, teenagers under 18 years of age):* Patients will be excluded per the study protocol. If logistical and scientific concerns can be adequately addressed, we will submit a revised application with an ethical framework, scientific support, and logistical considerations.

21.6 *Cognitively Impaired Adults, or adults with fluctuating or diminished capacity to consents:* To establish capacity in this setting, the MacArther Competence Assessment tool will be administered by trained study personnel. If the patient lacks capacity, a LAR may provide consent for these participants on their behalf. For adults unable to consent, consent will be obtained from a legally authorized representative in the following order of priority consistent with Minnesota State Law: health care agent previously assigned via a health care power of attorney, spouse, parents, adult children, and then adult siblings. An incapacitated adult who has a court appointed guardian would require permission of the court for enrollment, and these participants will be excluded practically from the trial.

21.7 *Adults Unable to Consent:* The same process will be applied as in 21.6.

21.8 *Assent and dissent in adults with fluctuating or limited mental capacity:*

- *Assent*: If patients might have capacity, we use the MacArthur Competence Assessment Tool for Clinical Research. If they do not reach the pre-specified threshold for capacity, we seek from an LAR. We do not seek further assent, as these patients are frequently intubated, delirious, or medically sedated
- *Dissent*: However, even if the LAR consents to the trial, if the patient is awake and alert, but dissent, we will exclude the patient.

22.0 Setting

22.1 *Research Sites:*

- Participating study sites will undergo a standardized screening process to ensure they can participate in necessary aspects of the study protocol, and comply with safety and remote oversight, legal, and regulatory procedures. All sites will cede to the central (Advarra) IRB in addition to undergoing local context review, as appropriate.

22.2 *International Research:* N/A

23.0 Multi-Site Research

23.1 *Study-Wide Number of Participants:* 200 minimum, up to 220 including screen failures

23.2 *Study-Wide Recruitment Methods:* N/A

23.3 *Study-Wide Recruitment Materials:* N/A

23.4 *Communication Among Sites:*

23.5 All sites have the most current version of the protocol, consent document(s), and, when applicable, HIPAA authorization.

- All required approvals (initial, continuing review, and modifications) have been obtained at each site (including by the site's IRB of record).
- All modifications have been communicated to sites, and approved (including approval by the site's IRB of record) before the modification is implemented.
- All engaged participating sites will safeguard data, including secure transmission of data, as required by local information security policies.
- All local site investigators conduct the study in accordance with applicable federal regulations and local laws.
- All non-compliance with the study protocol or applicable requirements will be reported in accordance with university or local policy.
- All other reportable events in accordance with university or local policy

23.6 *Communication to Sites:* Personnel from all sites will participate in monthly progress meeting calls, which will include discussion of any problems including reportable events, interim results, and completion of the study. More frequent communications may be planned, as becomes necessary.

24.0 Coordinating Center Research: N/A

25.0 Resources Available

25.1 ***Resources Available:*** It is difficult to estimate how many patients will develop COVID-19 in Minneapolis; however, given estimates from other countries it is likely to be 1000's if the virus is not contained.

The study PIs and research team will contribute at least 20% of their research time towards conducting and completing this study, we will leverage the rich resources of the UMN SIREN network hub and HCMC emergency department research infrastructure (of note, Dr. Puskarich is research director at HCMC, as well as associate research director at the University of Minnesota). In total across sites, this include >8 FTE of highly trained research nurses, coordinators, and research assistants across sites managed by the co-I plus an additional on-call pool of 8 additional trained associates providing 24/7 coverage for this and other studies. All study members have completed all required responsible conduct in research coursework and have extensive research experience. The research team is built for mobile, multi-site enrollment in time-sensitive emergency care research, and has been a consistent top enroller in the NIH supported emergency care SIREN, StrokeNET, NETT, and PETAL clinical trial networks for years. Additional expansion sites will undergo additional screening and training prior to expansion to ensure they are capable of executing the protocol as described.

26.0 References

1. Dong E, Du H, Gardner L. An interactive web-based dashboard to track COVID-19 in real time. *Lancet Infect Dis.* 2020.
2. John Hopkins C. Coronavirus COVID-19 (2019-nCoV). 2020; https://www.arcgis.com/apps/opsdashboard/index.html#/bda7594740fd40299423467b48e9ecf6.
3. World Health Organization. Novel Coronavirus (2019-nCoV) situation reports. 2020; https://www.who.int/emergencies/diseases/novel-coronavirus-2019/situation-reports.
4. Wang Y, Wang Y, Chen Y, Qin Q. Unique epidemiological and clinical features of the emerging 2019 novel coronavirus pneumonia (COVID-19) implicate special control measures. *J Med Virol.* 2020.
5. Zhao S, Lin Q, Ran J, et al. Preliminary estimation of the basic reproduction number of novel coronavirus (2019-nCoV) in China, from 2019 to 2020: A data-driven analysis in the early phase of the outbreak. *Int J Infect Dis.* 2020;92:214-217.
6. World Health O. Report of the WHO-China Joint Mission on Coronavirus Disease 2019 (COVID-19). In:2020.
7. Guan WJ, Ni ZY, Hu Y, et al. Clinical Characteristics of Coronavirus Disease 2019 in China. *N Engl J Med.* 2020.
8. Liu W, Tao ZW, Lei W, et al. Analysis of factors associated with disease outcomes in hospitalized patients with 2019 novel coronavirus disease. *Chin Med J (Engl).* 2020.

9. Zhang H, Penninger JM, Li Y, Zhong N, Slutsky AS. Angiotensin-converting enzyme 2 (ACE2) as a SARS-CoV-2 receptor: molecular mechanisms and potential therapeutic target. *Intensive Care Med.* 2020.
10. Severe Acute Respiratory Syndrome (SARS) and Coronavirus Testing—United States, 2003. *JAMA.* 2003;289(17):2203-2206.
11. de Wit E, van Doremalen N, Falzarano D, Munster VJ. SARS and MERS: recent insights into emerging coronaviruses. *Nature reviews Microbiology.* 2016;14(8):523-534.
12. Fang Y, Nie Y, Penny M. Transmission dynamics of the COVID-19 outbreak and effectiveness of government interventions: A data-driven analysis. *J Med Virol.* 2020.
13. Lu R, Zhao X, Li J, et al. Genomic characterisation and epidemiology of 2019 novel coronavirus: implications for virus origins and receptor binding. *Lancet.* 2020;395(10224):565-574.
14. Zhang L, Liu Y. Potential interventions for novel coronavirus in China: A systematic review. *J Med Virol.* 2020;n/a(n/a).
15. Gao J, Tian Z, Yang X. Breakthrough: Chloroquine phosphate has shown apparent efficacy in treatment of COVID-19 associated pneumonia in clinical studies. *Biosci Trends.* 2020;advpub.
16. Patel A, Jernigan DB, nCo VCDCRT. Initial Public Health Response and Interim Clinical Guidance for the 2019 Novel Coronavirus Outbreak - United States, December 31, 2019-February 4, 2020. *MMWR Morb Mortal Wkly Rep.* 2020;69(5):140-146.
17. Gurwitz D. Angiotensin receptor blockers as tentative SARS-CoV-2 therapeutics. *Drug Dev Res.* 2020.
18. Kickbusch I, Leung G. Response to the emerging novel coronavirus outbreak. *BMJ.* 2020;368:m406.
19. Sun ML, Yang JM, Sun YP, Su GH. [Inhibitors of RAS Might Be a Good Choice for the Therapy of COVID-19 Pneumonia]. *Zhonghua Jie He He Hu Xi Za Zhi.* 2020;43(0):E014.
20. Chen Y, Guo Y, Pan Y, Zhao ZJ. Structure analysis of the receptor binding of 2019-nCoV. *Biochem Biophys Res Commun.* 2020.
21. Imai Y, Kuba K, Rao S, et al. Angiotensin-converting enzyme 2 protects from severe acute lung failure. *Nature.* 2005;436(7047):112-116.
22. Kuba K, Imai Y, Rao S, et al. A crucial role of angiotensin converting enzyme 2 (ACE2) in SARS coronavirus-induced lung injury. *Nat Med.* 2005;11(8):875-879.
23. ClinicalTrials.Gov. Recombinant Human Angiotensin-converting Enzyme 2 (rhACE2) as a Treatment for Patients With COVID-19 - Full Text View - ClinicalTrials.gov. 2020; https://clinicaltrials.gov/ct2/show/NCT04287686.
24. Khan A, Benthin C, Zeno B, et al. A pilot clinical trial of recombinant human angiotensin-converting enzyme 2 in acute respiratory distress syndrome. *Crit Care.* 2017;21(1):234.
25. Li W, Moore MJ, Vasilieva N, et al. Angiotensin-converting enzyme 2 is a functional receptor for the SARS coronavirus. *Nature.* 2003;426(6965):450-454.
26. Glowacka I, Bertram S, Herzog P, et al. Differential downregulation of ACE2 by the spike proteins of severe acute respiratory syndrome coronavirus and human coronavirus NL63. *J Virol.* 2010;84(2):1198-1205.

27. Zou Z, Yan Y, Shu Y, et al. Angiotensin-converting enzyme 2 protects from lethal avian influenza A H5N1 infections. *Nat Commun.* 2014;5:3594.
28. Yang P, Gu H, Zhao Z, et al. Angiotensin-converting enzyme 2 (ACE2) mediates influenza H7N9 virus-induced acute lung injury. *Sci Rep.* 2014;4:7027.
29. Yan Y, Liu Q, Li N, et al. Angiotensin II receptor blocker as a novel therapy in acute lung injury induced by avian influenza A H5N1 virus infection in mouse. *Sci China Life Sci.* 2015;58(2):208-211.
30. Rice TW, Wheeler AP, Bernard GR, et al. Comparison of the SpO2/FIO2 ratio and the PaO2/FIO2 ratio in patients with acute lung injury or ARDS. *Chest.* 2007;132(2):410-417.
31. Pandharipande PP, Shintani AK, Hagerman HE, et al. Derivation and validation of Spo2/Fio2 ratio to impute for Pao2/Fio2 ratio in the respiratory component of the Sequential Organ Failure Assessment score. *Crit Care Med.* 2009;37(4):1317-1321.
32. Zhou FY, T. Du, R. Fan, G. Liu, Y. Liu Z, et al. Clinical Course and risk factors for mortality of adult inpatients with COVID-19 in Wuhan, China: a retrospective cohort study. Lancet, Epub Ahead of Print, March 11, 2020. .
33. Sica DA, Gehr TW, Ghosh S. Clinical pharmacokinetics of losartan. *Clin Pharmacokinet.* 2005;44(8):797-814.

Statistical Analysis Plan

ALPS-COVID Inpatient Trial

April 13, 2021

(Final draft prior to data analysis)

Statistical Analysis Plan

1. Introduction. 3
2. Trial Objectives. 3
3. Trial Design. 3
3.1. Randomization. 3
3.2. Sample size. 4
4. Study population. 5
4.1. Intent-to-treat. 5
4.2. Definition of Sub-Group Population in Different Analyses. 5
5. Trial Endpoints. 5
5.1. Primary Endpoint. 5
5.2. Secondary Endpoints. 7
5.3. Safety Endpoints. 9
5.4. Exploratory endpoints 9
6. Statistical Analysis. 10
6.1. General Approach. 10
6.2. Describing the Study Population. 10
6.3. Primary Endpoint Analysis. 10
6.3.1. Primary Analysis. 10
6.3.2. Secondary Analysis. 11
6.4. Secondary Endpoint Analysis. 11
6.5. Subgroup Analyses. 12
6.5. Safety. 12
6.5. Missing Data. 12
7. Statistical Analysis. 13

1. Introduction

This document will serve as the Statistical Analysis Plan for ALPS-COVID, Angiotensin receptor blocker based Lung Protective Strategy for COVID-19 Inpatient clinical trial. This is a two armed, phase II placebo controlled trial with patients allocated in a 1:1 ratio to losartan or placebo.

2. Trial Objectives

This phase II, blinded two-armed randomized control trial will test the efficacy of losartan compared to placebo to improve gas exchange as estimated by the estimated partial pressure of oxygen to fraction of inspired oxygen ratio (P/F). As few patients will have arterial blood gas measurements, the peripheral oxygen saturation (SaO2) will be utilized to estimate the P/F ratio. The imputation equations and assumptions are summarized below for the various potential modes of ventilation, devices, and assumptions regarding the fraction of inspired oxygen delivered by various approaches. Secondary outcomes are summarized below, but focus on an ordinal scale describing severity of illness by location at various time points, ventilator free days, oxygen free days, various adverse events, safety (particularly hypotension, vasopressor use, and kidney injury), and mortality. A subgroup analysis of biomarkers of the renin-angiotensin-aldosterone system will be conducted in patients with biospecimen analysis. Exploratory analyses are described. The primary analysis will be completed using intent to treat principles, an exploratory per-protocol analysis will be performed.

3. Trial Design

At the end of the Randomization Visit, participants (N ~ 200) will be randomized equally into one of two experimental conditions. Patients with an estimated GFR of >=60 will receive 50 mg losartan orally twice daily in suspension, while placebo patients will receive matched liquid. Those with eGFR 30-60 will receive once daily dosing. In the event of worsening eGFR, dosing will be decreased (from twice to once daily if eGFR decreases to 30-60, and stopped if eGFR <30). Patients will be treated with the intervention for 10 days or until hospital discharge, whichever comes first. Drug will not be continued after hospital discharge.

3.1. Randomization

Study subjects will be randomized with equal probability to one of the two groups defined by the two factors defined above. Randomization will be completed using permuted block randomization with random block sizes, stratified by enrollment site and age (>= or < 60). This cutoff was chosen based on early data suggesting markedly different outcomes by age, in order to mitigate potential unequal allocation to each arm. Randomization schedules will be generated using permuted blocks with randomly varying sizes of 2, 4 or 6 and stored in an Oracle database. We anticipate no or minimal block effects in our data and, therefore, we will not account for block effects in our data analysis.

3.2. Sample Size

We will enroll ~200 subjects (n = 100 / group). At the time of study design, the expected standard deviations of P/F ratios, inclusive of non-intubated patients, in COVID are unknown. Prior literature in acute lung injury and acute respiratory distress trials suggests a range of 75-125 in most changes. Assuming a fixed sample size of 200, we analyzed power using a number of assumptions, illustrated in the Figure below, calculated assuming t-tests with equal variance, a type I error rate of 0.05 and a sample size of 200. If the SD is 125 or less, we will have >0.8 power to detect a difference in P/F of 50. Differences of less than this are of uncertain clinical significance. This method and outcome has more power than an alternative approach of respiratory SOFA, which is a more crude measure of the estimated P/F ratio.

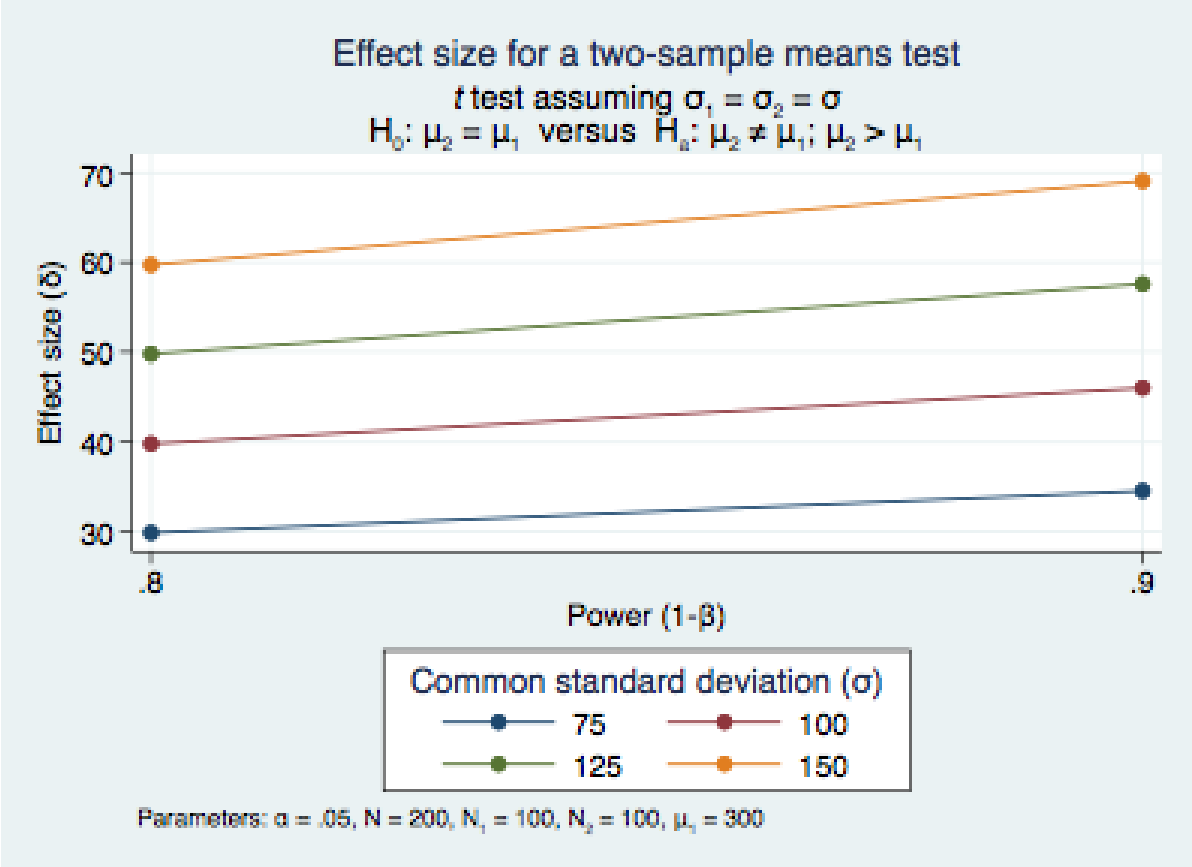

4. Study Populations

4.1. Intent-to-Treat

The primary analysis of all endpoints will adhere to the intent-to-treat principle. Under this principle, all randomized subjects will be included in the analysis in the group to which they were randomized regardless of protocol violations and compliance to treatment assignment.

An exploratory per-protocol analysis will be conducted of patients who received at least 2 days of their assigned treatment allocation.

4.2. Definition of Sub-Group Population in Different Analyses

We intend to complete pre-planned subgroup analyses by assigned sex, age group (<60, >=60), patients with a past medical history of hypertension, eGFR at enrollment (<60, >= 60), and those with a systolic blood pressure of >=135 at the time of enrollment. Subgroup analysis will allow us to evaluate the consistency of the effect of losartan across important subgroups. Furthermore, as hypertension history and blood pressure may be a surrogate of renin-angiotensin-aldosterone system (RAAS) activation, this may be a clinically useful way to bring a precision medicine paradigm to RAAS activation in COVID-19.

Biomarker analyses will be conducted on the *de facto* subgroup with biospecimen availability who had relevant biomarkers analyzes.

5. Trial Endpoints

5.1. Primary Endpoint

Difference in Estimated (PEEP adjusted) P/F Ratio at 7 days [Time Frame: 7 days]

Outcome calculated from the partial pressure of oxygen or peripheral saturation of oxygen by pulse oximetry divided by the fraction of inspired oxygen (PaO2 or SpO2 : FiO2 ratio or P/F ratio). PaO2 is preferentially used if available, otherwise peripheral oxygen saturation will be used to estimate the P/F ratio. A correction is applied for endotracheal intubation and/or positive end-expiratory pressure. Patients receiving non-invasive oxygen will have P/F estimated using the equation below. Assumptions regarding fraction of inspired oxygen for patients not receiving a set FiO2 will be applied uniformly using tables below. Patients discharged prior to day 7 will have a home pulse oximeter sent home for measurement of the day 7 value, and will be adjusted for home O2 use, if applicable. Patients who died will be applied a penalty, with a P/F ratio of 0. The worst daily values will be utilized.

Method of P/F estimation:

1. If partial pressure of oxygen (PaO2) available: P/F = PaO2 / FiO2

2. If on positive pressure ventilation with no PaO2 available, by positive end expiratory pressure (PEEP), where SpO2 is in decimal form.

$$\widehat{PF} = 10^{0.06+0.94*log_{10}(\frac{SpO_2*100}{FiO_2})} \text{ for } PEEP < 8$$
$$\widehat{PF} = 10^{-0.13+1.01*log_{10}(\frac{SpO_2*100}{FiO_2})} \text{ if } 8 \leq PEEP \leq 12$$
$$\widehat{PF} = 10^{-0.47+1.17*log_{10}(\frac{SpO_2*100}{FiO_2})} \text{ if } PEEP > 12.$$

3. If on supplemental oxygen with no PaO2 available, where SpO2 is in decimal form.

$$\widehat{PF} = \frac{PaO_2}{FiO_2} = \frac{(\frac{23400}{\frac{1}{SpO_2}-0.99})^{1/3}}{FiO_2}.$$

Assumptions for FiO2 if not receiving mechanical ventilation:

If supplemental oxygen delivered via low-flow cannula:

a. If flow <=8 Liters per minute,
   i. FiO2 estimated with 0.21 + (0.03 * flow rate)
b. If flow 8-15 Liters per minute,
   i. FiO2 estimated with 0.45 + [0.02 * (flow rate – 8)]

As supplemental oxygen delivered via facemask with flow rates of <=8 are typically delivered with a simple mask, and those >8 with non-rebreather:

c. If 4<= flow rate <= 8 Liters per minutes,
   i. FiO2 estimated = 0.4 + [0.05 * (flow rate - 4)]
d. If 8 < flow rate <=9.5 Liters per minute,
   i. FiO2 estimated with 0.1 * flow rate
e. If flow rate >9.5,
   i. FiO2 estimated with 0.95
f. For flow rates <4, the equations for nasal cannula will be used

If supplemental oxygen delivered via high flow nasal cannula, the following table is utilized with an assumed tidal volume (Vt) of 500 mL.

Table 2

Set F_{IO_2}	Flow (L/min)	V_T (mL) 300	500	700
0.3	20	0.29 ± 0	0.27 ± 0.01	0.26 ± 0.01
	40	0.30 ± 0	0.30 ± 0	0.29 ± 0.01
	60	0.31 ± 0	0.31 ± 0	0.31 ± 0
0.5	20	0.46 ± 0.02	0.40 ± 0.03	0.36 ± 0.02
	40	0.49 ± 0.02	0.48 ± 0.01	0.45 ± 0.02
	60	0.50 ± 0	0.50 ± 0	0.50 ± 0
0.7	20	0.62 ± 0.02	0.53 ± 0.04	0.46 ± 0.03
	40	0.69 ± 0	0.66 ± 0.01	0.61 ± 0.04
	60	0.70 ± 0	0.69 ± 0.01	0.69 ± 0.01

For set FiO2s and set Flow rates between values listed on the table, linear interpolation is utilized, while linear extrapolation from the two closest points are used for values outside the table (e.g. set FiO2 = 1.0)

5.2. Secondary Endpoints

1. Difference in estimated P/F ratio over time using generalized estimating equation
2. Sequential Organ Failure Assessment (SOFA) Total Score [Time Frame: 10 days]
   The SOFA assessment is used to track a person's risk status during stay in the Intensive Care Unit (ICU). The score is based on six different scores, one each for the respiratory, cardiovascular, hepatic, coagulation, renal, and neurological systems. Each organ system is assigned a point value from 0 (normal) to 4 (high degree of dysfunction/failure). Total score is calculated by entering patient data into a SOFA calculator, a widely-available software. Total scores range from 0-24, with higher scores indicating greater chance of mortality.
3. Oxygen Saturation / Fractional Inhaled Oxygen (S/F) [Time Frame: 10 days]
   Oxygen saturation (percent) is measured by pulse oximeter. Fraction of inspired oxygen (FiO2) (unitless) is the volumetric fraction of oxygen to other gases in respiratory support. The S/F ratio is unitless.
4. 28-Day Mortality [Time Frame: 28 days]
   Outcome reported as the number of participants who have expired at 28 days post enrollment.

5. 90-Day Mortality [Time Frame: 90 days]
   Outcome reported as the number of participants who have expired at 90 days post enrollment.
6. ICU Admission [Time Frame: 10 days]
   Outcome reported as the number of participants in each arm who require admission to the Intensive Care Unit (ICU).
7. Number of Ventilator-Free Days [Time Frame: 10 days]
   Outcome reported as the mean number of days participants in each arm did not require mechanical ventilation during an in-patient hospital admission.
8. Number of Therapeutic Oxygen-Free Days [Time Frame: 10 days]
   Outcome reported as the mean number of days participants in each arm did not require therapeutic oxygen usage during an in-patient hospital admission.
9. Number of Vasopressor-Free Days [Time Frame: 10 days]
   Outcome reported as the mean number of days participants in each arm did not require vasopressor usage during an in-patient hospital admission.
10. Length of ICU Stay [Time Frame: 10 days]
    Outcome reported as the mean length of stay (in days) in the Intensive Care Unit (ICU) for participants in each arm.
11. Length of Hospital Stay [Time Frame: 10 days]
    Outcome reported as the mean length of in-patient hospital stay (in days) for participants in each arm.
12. Incidence of Respiratory Failure [Time Frame: 10 days]
    Outcome reported as the number of participants requiring BiPAP OR high flow nasal cannula OR mechanical ventilation OR extracorporeal membranous oxygenation (ECMO) utilization during in-patient hospital care in each arm.
13. Change in PROMIS Dyspnea scale [Time Frame: 10 days]
    The PROMIS Dyspnea (shortness of breath) item banks and pools assess self-reported shortness of breath in general, intensity, frequency and duration on a 0-10 scale, with 0 being no symptoms and 10 being the most severe. Finally, the patient answers the question "I've been short of breath" using a 0-4 scale, 0 being none and the most severe. There is no validated, unified single score and each item is evaluated individually.
14. Change in SF-12 Physical Composite Score [Time Frame: 10 days]
    The SF-12 is a self-reported validated outcome measure assessing the impact of health on an individual's everyday life. Patients fill out a 12 question survey which is then scored by a clinician or researcher. Physical score is computed using the scores of twelve questions and range from 0 to 100, where a zero score indicates the lowest level of health measured by the scales and 100 indicates the highest level of health.
15. Change in SF-12 Mental Composite Score [Time Frame: 10 days]
    The SF-12 is a self-reported validated outcome measure assessing the impact of health on an individual's everyday life. Patients fill out a 12 question survey which is then scored

by a clinician or researcher. Mental composite score is computed using the scores of twelve questions and range from 0 to 100, where a zero score indicates the lowest level of health measured by the scales and 100 indicates the highest level of health.

16. Disease Severity Rating [Time Frame: 10 days]
    Outcome reported as the number of participants in each arm who fall into each of 7 categories. Lower scores indicate greater condition severity. The ordinal scale is an assessment of the clinical status at the first assessment of a given study day. The scale is as follows: 1) Death; 2) Hospitalized, on invasive mechanical ventilation or extracorporeal membrane oxygenation (ECMO); 3) Hospitalized, on non-invasive ventilation or high flow oxygen devices; 4) Hospitalized, requiring supplemental oxygen; 5) Hospitalized, not requiring supplemental oxygen; 6) Not hospitalized, limitation on activities; 7) Not hospitalized, no limitations on activities.
17. Viral Load by Nasopharyngeal Swab Day 9 [Time Frame: 9 days]
    Nasopharyngeal swabs will be collected every third day for the duration of study participation. Viral load is measured as number of viral genetic copies per mL.
18. Viral Load by Nasopharyngeal Swab Day 15 [Time Frame: 15 days]
    Nasopharyngeal swabs will be collected every third day for the duration of study participation. Viral load is measured as number of viral genetic copies per mL.
19. Viral Load by Blood Day 9 [Time Frame: 9 days]
    Blood will be collected every third day for viral load assessment for the duration of study participation. Viral load is measured as number of viral genetic copies per mL.
20. Viral Load by Blood Day 15 [Time Frame: 15 days]
    Blood will be collected every third day for viral load assessment for the duration of study participation. Viral load is measured as number of viral genetic copies per mL.

5.3. Safety Endpoints

1. Adverse events
2. Serious adverse events
3. Daily Hypotensive Episodes [Time Frame: 10 days]
   Outcome reported as the mean number of daily hypotensive episodes (MAP < 65 mmHg) prompting intervention (indicated by a fluid bolus >=500 mL) per participant in each arm.
4. Hypotension Requiring Vasopressors [Time Frame: 10 days]
   Outcome reported as the number of participants in each arm requiring the use of vasopressors for hypotension.
5. Acute Kidney Injury [Time Frame: 10 days]
   Outcome reported as the number of participants in each arm who experience acute kidney

injury as defined by the Kidney Disease Improving Global Outcomes (KDIGO) guidelines:
Increase in serum creatinine by 0.3mg/dL or more within 48 hours OR Increase in serum creatinine to 1.5 times baseline or more within the last 7 days OR Urine output less than 0.5 mL/kg/h for 6 hours.

5.4 Exploratory endpoints

1. Angiotensin II (ang II), angiotensin 1-7 (ang 1-7), and ratio of Ang II/ang 1-7
2. Modification of treatment effect by pre-enrollment measurements of, ang II and DNA methylation

6. Statistical Analysis

6.1. General Approach

All statistical analyses will be performed using SAS (version 9.4 or newer) or R (version 4.0.3 or newer). All statistical tests will be two-tailed with P-values less than 0.05 considered statistically significant unless otherwise noted. All analyses will be completed using the intent-to-treat principle unless otherwise noted. Methods for handling missing data will be specified below.

6.2. Describing the Study Population

Baseline covariates will be summarized by treatment group to identify any treatment group imbalances post randomization. This will include location of enrollment, demographic characteristics (age, sex, race/ethnicity, bmi, insurance type), and comorbid conditions, including CAD, hypertension, CHF, Asthma, COPD, Bronchitis, OSA, Diabetes, and tobacco use history. Continuous covariates will be summarized by the mean (standard deviation) or median (25, 75 percentiles) when skewed. Categorical and binary covariates will be summarized by frequency (percentage).

Baseline vitals and symptoms will be summarized by treatment group similarly. Vitals will include Time Before Seeking Care, Oxygen Requirements, SBP, DBP, MAP, Temperature, Heart rate, Respiratory rate, Oxygen saturation, Fi02, PEEP, Lymphocytes, Neutrophils, CRP, White Blood Cell count, Platelets, Creatinine, Potassium, AST, ALT and Total Bilirubin. Symptoms will include Fever, Cough (by Type), Sore Throat, Rhinorrhea, Wheezing, Chest Pain, Myalgias, Joint Pain, Abdominal Pain, Malaise, Shortness of Breath, Inability to walk. Headache, Altered Mental Status. Seizures, Nausea, Diarrhea, Conjunctivitis, Skin rash, Skin ulcers. Lymphadenopathy, Hemorrhage, Loss of smell. Loss of taste, Sinus congestion, Other symptoms.

6.3. Primary Endpoint Analysis

The primary endpoint is the P/F ratio on Study Day 7 with participants who have expired by Day 7 receiving a P/F ratio of 0.

6.3.1. Primary Analysis

The objective of our primary analysis is to test the main effect of losartan on P/F ratio on Day 7. We will do this by fitting a linear regression model with a main effect for losartan (versus placebo) and linear adjustment for baseline P/F ratio. The statistical significance of the main effect will be evaluated using a likelihood ratio test, carried out by fitting the smaller nested linear regression model that omits the main effect term but includes the adjustment for baseline P/F ratio. The adjustment for baseline P/F ratio is intended to reduce residual variance and thereby improve the power for detecting a statistically significant main effect. Assumptions about normality and homoscedasticity will be evaluated by plotting histograms of the residuals by treatment group. Transformations of the outcome variable and a robust sandwich variance estimator may be considered if violations of model assumptions are observed.

6.3.2. Secondary Analysis

A secondary unadjusted analysis of the primary outcome will be carried out using Welch's t-test. Transformations of the outcome variable and a non-parametric Wilcoxon rank-sum test may be considered if violations of the normality assumptions are observed.

Exploratory analyses considering other baseline adjusters that appear imbalanced between study arms (p-value less than 0.20) and are putatively related to the outcome may be undertaken as well.

P/F ratio on Study Days 1 - 10 will be analyzed using generalized estimating equations with an identity link and AR1 working correlation matrix assumption. These GEE analyses will include similar sets of adjusters.

In addition to the primary intention to treat analysis, a per-protocol analysis will be carried out including participants who took at least 2 days worth of their assigned study drug.These analyses will be analogous to the intention to treat analyses but on this modified per-protocol subgroup.

6.4. Secondary Endpoint Analyses

Secondary endpoints will be analyzed following the same approach as the primary endpoint, particularly S/F ratio on Study Day 7. Secondary endpoints that are collected longitudinally will be analyzed using generalized linear mixed-models or generalized estimating equations, as appropriate, adjusting only for the corresponding baseline measurement when available. In particular, viral load data will be analyzed using linear mixed models with a visit by treatment group interaction and a global Wald test for testing overall statistical significance of the treatment. Both mean cycle threshold and

relative viral load will be analyzed as outcome variables with undetected results assigned a value of 45, which reflects the maximum cycle threshold.

Overall mortality will be evaluated using Kaplan-Meier plots and the log-rank test with a hazard ratio estimate based on an unadjusted Cox model. Adjusted Cox models will be implemented with adjusted included similarly to the primary analysis. Time to hospital discharge, with death in hospital or discharge to comfort care as a competing risk, will be evaluated using cumulative incidence plots and Fine-Gray's test (rho = 0), which is the competing risks analog to the log-rank test. Unadjusted competing risks regression will be used to estimate the recovery rate ratio, and adjusted models will be considered similarly to the primary analysis.

Secondary endpoints that are collected at a single time-point will be analyzed using an appropriate regression framework (i.e. linear, logistic, etc.) or an exact framework for binary outcomes (i.e. unconditional exact tests and exact confidence intervals) when appropriate. Ordinal outcomes (i.e., NIH disease severity scale) will be analyzed using proportional odds regression.

6.5. Subgroup Analyses

Subgroup analyses will follow the same approach as was described above within each subgroup. As outlined in 5.2 above, we intend to complete pre-planned subgroup analyses by assigned sex, age group (<60, >=60), patients with a past medical history of hypertension, those with a systolic blood pressure of >=135 at the time of enrollment, and eGFR at enrollment (<60, >= 60), but other subgroups may also be considered. Biomarker analyses will be conducted on the *de facto* subgroup with biospecimen availability who had relevant biomarker analyses.

Formal testing of the interaction between subgroup and treatment effect is not the primary concern of our subgroup analyses. Instead, subgroup analyses will provide information about the consistency of the treatment effect across subgroups, which will provide supplementary information for full understanding of the relationship between the studied interventions and the study endpoints. We will not use formal multiple-comparison adjustments for our subgroup analyses as they are considered exploratory.

6.6. Safety

AEs and SAEs will be tabulated and rates over during the initial 10 Study Days will be compared across treatment groups using negative binomial regression. Participants who withdraw prior to Day 10 will contribute partial information to these analyses. Daily hypertensive episodes, hypotension requiring vasopressors, and acute kidney injuries will be tabulated and analyzed similarly.

6.7. Missing Data

An intent-to-treat analysis requires that subjects are randomized according to their randomized treatment assignment, regardless of compliance to the study product, and that complete data are available on all study subjects. Every effort will be made to limit the amount of missing data in this trial. However, some level of missing data is inevitable. In

response, we will complete a sensitivity analysis for the primary endpoint in order to evaluate the robustness of our conclusions to missing data.

We will compare subjects with and without missing data in order to identify baseline covariates associated with missing data. Our primary approach to handling missing primary endpoint data on Day 7 will be to conduct our analysis using multiple imputation. Specifically, we will use predictive mean matching to multiply impute missing outcomes with stratification by hospitalization status. In particular, missing outcome data for participants who were discharged on Day 7 will be imputed based on the observed outcomes for participants who were also discharged on Day 7. Imputation regression models will be developed based on all available data. The results of these analyses will be compared to the primary analysis to evaluate the robustness of our conclusions.

References

Chikata Y, Onodera M, Oto J, Nishimura M. FIO2 in an Adult Model Simulating High-Flow Nasal Cannula Therapy. Respir Care. 2017 Feb;62(2):193-198. doi: 10.4187/respcare.04963. Epub 2016 Nov 22. PMID: 27879385.

Pandharipande PP, Shintani AK, Hagerman HE, St Jacques PJ, Rice TW, Sanders NW, Ware LB, Bernard GR, Ely EW. Derivation and validation of Spo2/Fio2 ratio to impute for Pao2/Fio2 ratio in the respiratory component of the Sequential Organ Failure Assessment score. *Crit Care Med*. 2009 Apr;37(4):1317-21. doi: 10.1097/CCM.0b013e31819cefa9. PMID: 19242333; PMCID: PMC3776410.

Shrirang M Gadrey, Chelsea E Lau, Russ Clay, Garret T Rhodes, Douglas E Lake, Christopher C Moore, John D Voss and J Randall Moorma. Imputation of partial pressures of arterial oxygen using oximetry and its impact on sepsis diagnosis. 2019 Institute of Physics and Engineering in Medicine. Physiological Measurement, Volume 40, Number 11
