# Supplementary material for: Efficacy of Losartan in Hospitalized Patients With COVID-19–Induced Lung Injury: A Randomized Clinical Trial
Source: JAMA Netw Open. 2022 Mar 16;5(3):e222735. doi: 10.1001/jamanetworkopen.2022.2735 (PMC8928006; doi:10.1001/jamanetworkopen.2022.2735)
Supplement: Supplement 2. — eTable 1. Pao2:Fio2 Ratio at Baseline With Day 7 Results eTable 2. Inpatient Nonfatal Adverse Events Per Person-Day, Days 1-15 eTable 3. Losartan and Carboxylosartan Pharmacokinetic Characteristics eTable 4. Association Between Treatment Group and Circulatory RAAS Components After Adjusting for Baseline and Time and Jointly Modeling the Outcome and Time-to-Discharge eFigure 1. Effect of Losartan on Relative Viral Load eFigure 2. Losartan and Carboxylosartan Concentrations eFigure 3. Plasma Log(AII) From Days 1-15, by Treatment Allocation eFigure 4. Plasma Log(angiotensin-[1-7]) From Days 1-15, by Treatment Allocation [file jamanetwopen-e222735-s002.pdf]

Supplemental Online Content

Puskarich MA, Ingraham NE, Merck LH, et al; Angiotensin Receptor Blocker Based Lung Protective Strategies for Inpatients With COVID-19 (ALPS-IP) Investigators. Efficacy of losartan in hospitalized patients with COVID-19–induced lung injury: a randomized clinical trial. *JAMA Netw Open*. 2022;5(3):e222735. doi:10.1001/jamanetworkopen.2022.2735

eTable 1. PaO_2:FiO_2 Ratio at Baseline With Day 7 Results
eTable 2. Inpatient Nonfatal Adverse Events Per Person-Day, Days 1-15
eTable 3. Losartan and Carboxylosartan Pharmacokinetic Characteristics
eTable 4. Association Between Treatment Group and Circulatory RAAS Components After Adjusting for Baseline and Time and Jointly Modeling the Outcome and Time-to-Discharge
eFigure 1. Effect of Losartan on Relative Viral Load
eFigure 2. Losartan and Carboxylosartan Concentrations
eFigure 3. Plasma Log(AII) From Days 1-15, by Treatment Allocation
eFigure 4. Plasma Log(angiotensin-[1-7]) From Days 1-15, by Treatment Allocation

This supplemental material has been provided by the authors to give readers additional information about their work.

© 2022 Puskarich MA et al. *JAMA Network Open.*

eTable 1. PaO_2:FiO_2 Ratio at Baseline With Day 7 Results

Reported quantities reflect Median (IQR) or N (%).		
	**Placebo** (n = 104)	**Losartan** (n = 101)
P/F Ratio at Baseline	214.1 [138.4 - 253.4]	214.1 [153.6 - 258.4]
Discharged on or before Day 7	62 (59.6%)	49 (48.5%)
Hospitalized through Day 7	39 (37.5%)	50 (49.5%)
Dead on or before Day 7	3 (2.9%)	2 (2.0%)
P/F Ratio Missing on Day 7 (Alive)	16 (15.4%)	10 (9.9%)

© 2022 Puskarich MA et al. *JAMA Network Open.*

eTable 2. Inpatient Nonfatal Adverse Events Per Person-Day, Days 1-15

	Losartan		Placebo		
	**Number of Events**	**Rate (SD)**	**Number of Events**	**Rate (SD)**	**P-value**
**Adverse events (including serious adverse events)**					
Cardiovascular	26	0.021 (0.053)	24	0.021 (0.077)	0.96
Endocrine	2	0.001 (0.010)	1	0.001 (0.007)	0.51
Ear, Nose, and Throat	0	0.000 (0.000)	1	0.001 (0.007)	0.32
Gastrointestinal	10	0.007 (0.021)	3	0.002 (0.011)	0.04
Genitourinary	3	0.002 (0.011)	1	0.002 (0.016)	0.84
Hematologic	9	0.006 (0.022)	6	0.005 (0.028)	0.72
Musculoskeletal	0	0.000 (0.000)	0	0.000 (0.000)	
Neurologic	4	0.003 (0.013)	4	0.003 (0.016)	0.97
Renal	20	0.014 (0.051)	21	0.017 (0.055)	0.68
Respiratory	30	0.023 (0.051)	27	0.023 (0.064)	0.91
Skin	0	0.000 (0.000)	2	0.001 (0.009)	0.16
Total	104	0.077 (0.154)	90	0.074 (0.204)	0.89
**Serious Adverse Events Only**					
Cardiovascular	14	0.011 (0.035)	6	0.005 (0.023)	0.16
Endocrine	0	0.000 (0.000)	0	0.000 (0.000)	
Ear, Nose, and Throat	0	0.000 (0.000)	0	0.000 (0.000)	
Gastrointestinal	1	0.001 (0.007)	0	0.000 (0.000)	0.32
Genitourinary	0	0.000 (0.000)	0	0.000 (0.000)	

© 2022 Puskarich MA et al. *JAMA Network Open.*

Hematologic	1	0.001 (0.007)	0	0.000 (0.000)	0.32
Musculoskeletal	0	0.000 (0.000)	0	0.000 (0.000)	
Neurologic	2	0.001 (0.009)	1	0.001 (0.007)	0.55
Renal	1	0.001 (0.007)	2	0.002 (0.013)	0.47
Respiratory	19	0.016 (0.044)	21	0.019 (0.063)	0.75
Skin	0	0.000 (0.000)	0	0.000 (0.000)	
Total	38	0.030 (0.069)	30	0.026 (0.082)	0.68

© 2022 Puskarich MA et al. *JAMA Network Open.*

eTable 3. Losartan and Carboxylosartan Pharmacokinetic Characteristics

**Study ID**		**Losartan**							**Carboxylosartan (LCA)**		
	**Dose (mg)**	**Half-Life (hr)**	**C_{max} (ng/mL)**	**C_{6h} (ng/mL)**	**est C_{12h} (ng/mL)**	**AUC_{0-6h} (hr*ng/mL)**	**AUC_{0-12h} (hr*ng/mL)**	**CL/F (L/hr)**	**C_{max}**	**C_{6h} (ng/mL)**	**AUC_{0-6h} (hr*ng/mL)**
1	50	2.87	268.6	102.2	22.98	1018.0	1336.5	37.41	16.5	16.5	66.6
2	50	1.53	1029	168.8	11.22	3101.3	3450.0	14.49	263.1	263.1	1206.2
3	50	1.56	145.6	24.76	1.77	449.7	501.9	99.61	357.7	205.7	1803.6
4	50	1.77	217.9	45.38	3.99	657.9	760.0	65.79	129.2	113.6	578.0
5	50	5.27	131.4	101	45.87	565.0	984.0	50.81	164.8	164.8	375.4
6	50	2.45	110.8	35.76	6.27	395.3	496.9	100.61	293.5	293.5	1114.8
7	50	1.21	586.4	59.14	1.78	1493.2	1591.5	31.42	667.1	338.5	2771.4
**Median**		1.77	217.9	59.1	6.3	657.9	984.0	50.8	263.1	205.7	1114.8
**Min**		1.21	110.8	24.8	1.8	395.3	496.9	14.5	16.5	16.5	66.6
**Max**		5.27	1029.0	168.8	45.9	3101.3	3450.0	100.6	667.1	338.5	2771.4

C_{max}, maximum concentration in plasma; C_{6h}, measured concentration at 6 hours post dose; est C_{12h}, estimated concentration at 12 hours post dose; AUC_{0-6h}, area under the concentration time curve to 6 hours post dose; AUC_{0-12h}, estimated area under the concentration time curve to 12 hours post dose; CL/F, apparent oral clearance.

© 2022 Puskarich MA et al. *JAMA Network Open.*

eTable 4. Association Between Treatment Group and Circulatory RAAS Components After Adjusting for Baseline and Time and Jointly Modeling the Outcome and Time-to-Discharge

Death treated as censored. Significant p values for log(baseline) indicate significant association of baseline measurements with later measurements, after controlling for treatment allocation and time.

**Covariate**	**ACE**		**ACE2**		**Ang-(1–7)**		**Ang II**	
	Ratio of Geometric Mean	p-value	Ratio of Geometric Mean	p-value	Ratio of Geometric Mean	p-value	Ratio of Geometric Mean	p-value
**Losartan**	0.90 (0.72, 1.13)	0.39	1.17 (0.71, 1.92)	0.54	0.99 (0.63, 1.54)	0.95	1.07 (0.76, 1.50)	0.72
**Log (baseline)**	2.73 (2.37, 3.13)	<0.01	2.15 (1.87, 2.47)	<0.01	1.71 (1.44, 2.05)	<0.01	1.62 (1.23, 2.15)	<0.01
**Day**	1.02 (1.00, 1.04)	0.12	1 (0.97, 1.04)	0.98	0.98 (0.94, 1.01)	0.16	1 (0.97, 1.03)	0.96

ACE - Angiotensin-converting enzyme activity; ACE 2 - Angiotensin-converting enzyme 2 activity; Ang-(1–7) - angiotensin-(1–7) concentration; Ang II - angiotensin II concentration

eFigure 1. Effect of Losartan on Relative Viral Load
Relative viral load (RVL) is corrected to human marker DNA to control for specimen quality. Placebo is in red lines and losartan in blue lines with 95% CIs at each assessment. Losartan did not statistically significantly affect the cycle threshold or relative viral load overall or at any time point.

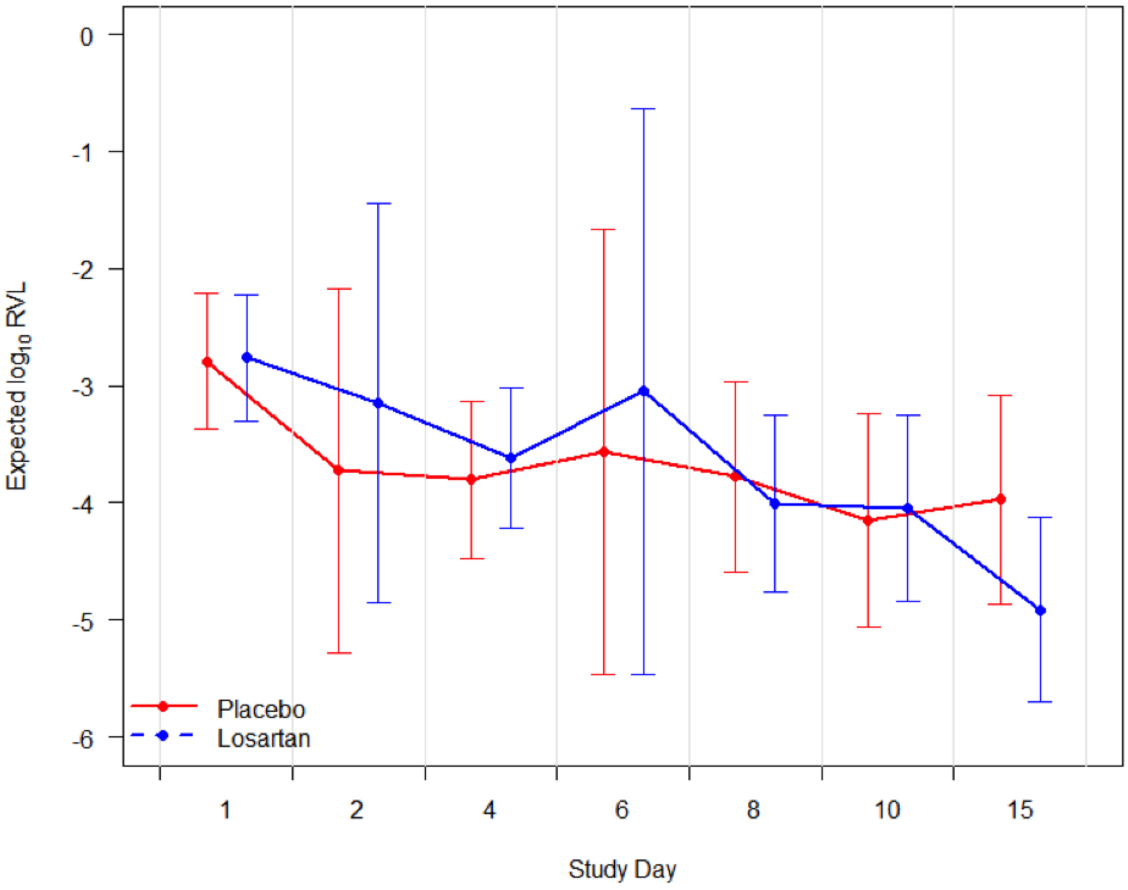

© 2022 Puskarich MA et al. *JAMA Network Open.*

eFigure 2. Losartan and Carboxylosartan Concentrations
(median and range)

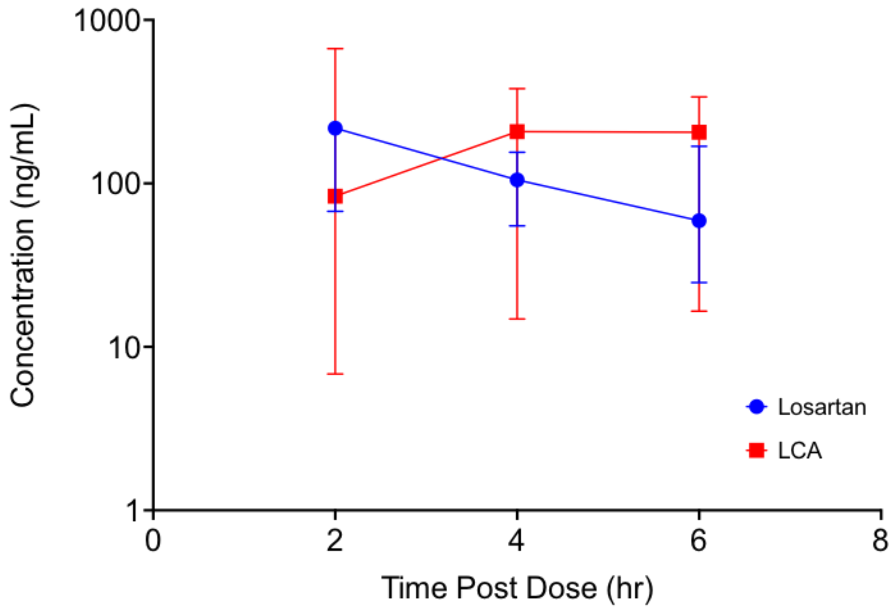

© 2022 Puskarich MA et al. *JAMA Network Open.*

eFigure 3. Plasma Log(AII) From Days 1-15, by Treatment Allocation
Participants with missing values are indicated with a red “x”, otherwise each participant’s values are connected with a grey line.

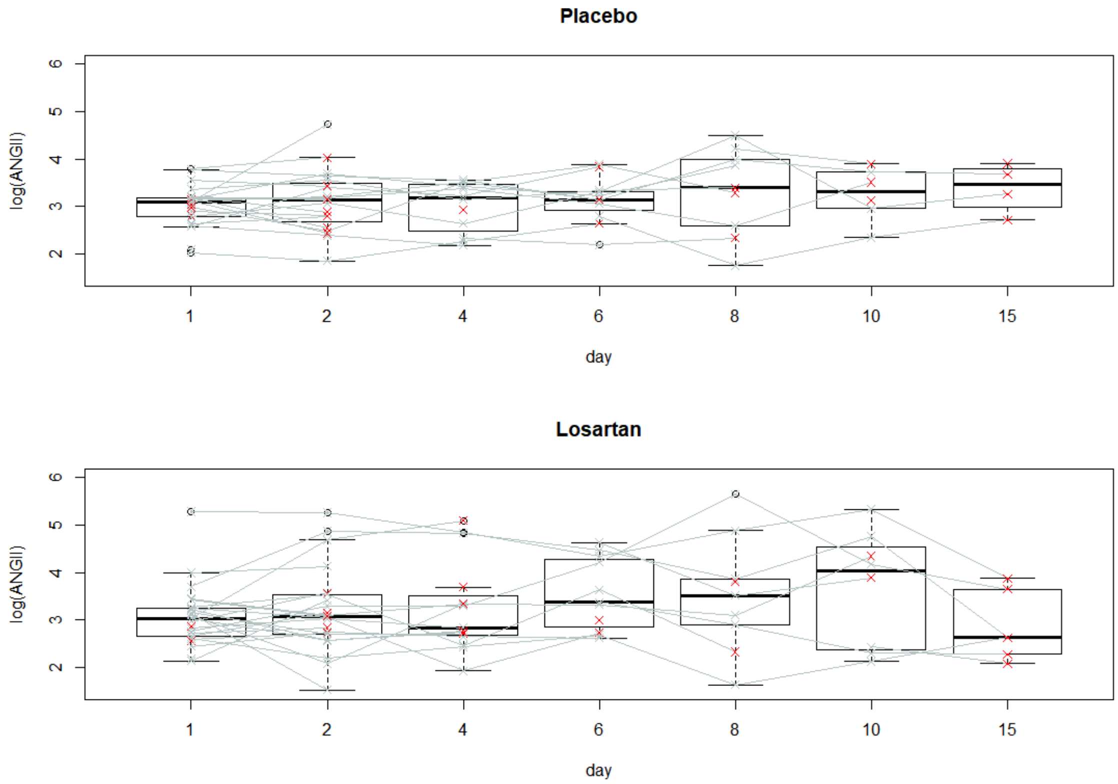

© 2022 Puskarich MA et al. *JAMA Network Open.*

eFigure 4. Plasma Log(angiotensin-[1-7]) From Days 1-15, by Treatment Allocation
Participants with missing values are indicated with a grey "x" when present and a red "x" if missing, connected with a grey line.

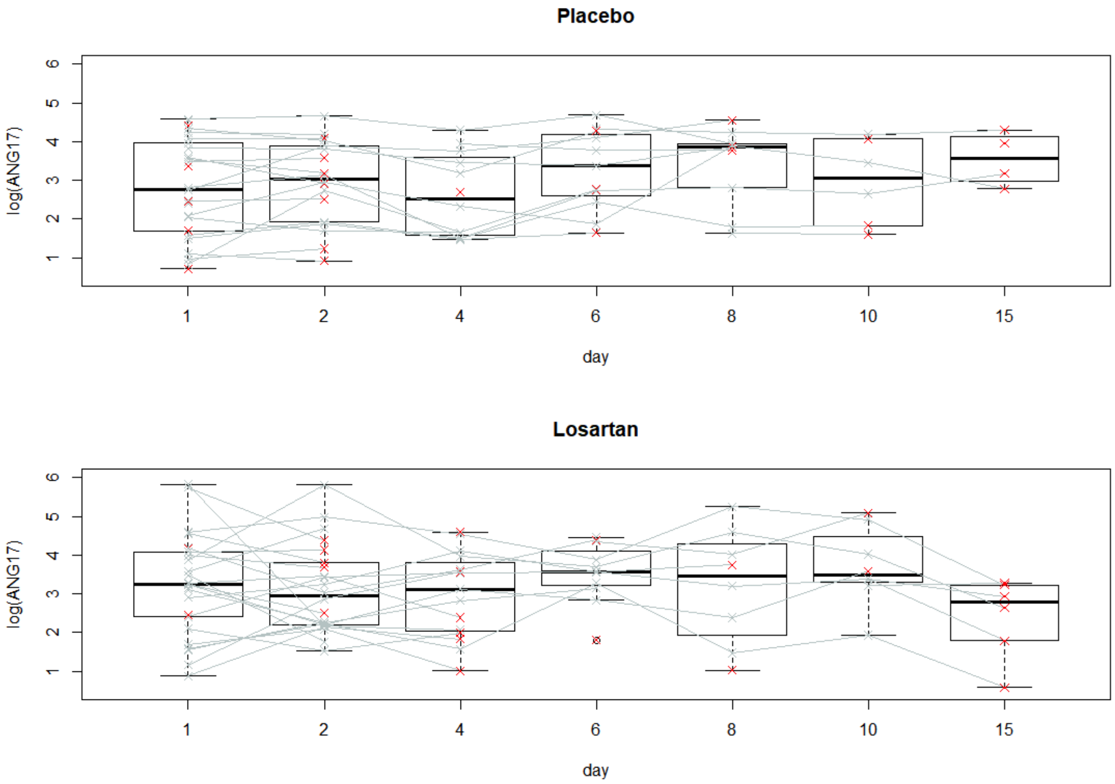

© 2022 Puskarich MA et al. *JAMA Network Open.*
