# Supplementary material for: Efficacy of Losartan in Hospitalized Patients With COVID-19–Induced Lung Injury: A Randomized Clinical Trial
Source: JAMA Netw Open. 2022 Mar 16;5(3):e222735. doi: 10.1001/jamanetworkopen.2022.2735 (PMC8928006; doi:10.1001/jamanetworkopen.2022.2735)
Supplement: Supplement 3. — Angiotensin Receptor Blocker Based Lung Protective Strategies for Inpatients With COVID-19 (ALPS-IP) Investigators [file jamanetwopen-e222735-s003.pdf]

Supplemental Online Content: Nonauthor Collaborators

*Indicates required information. Only first name, last name, and suffix will appear in PubMed.

| *Group Name(s): Angiotensin Receptor Blocker Based Lung Protective Strategies for Inpatients with COVID-19 (ALPS-IP) Investigators | | | | | | | |
|---|---|---|---|---|---|---|---|
| ***First Name and Middle Initial(s)** | ***Last Name** | ***Suffix (eg, Jr, III)** | Academic Degrees | Institution | Location (city, state/province, country) | Role or Contribution, eg, chair, principal investigator | Group (if more than 1 Group listed in the byline) and/or Subgroup (eg, Steering Committee) |
| Andrew C | Nelson | | MD PhD | Department of Laboratory Medicine and Pathology, University of Minnesota | Minneapolis, MN, USA | Oversaw Viral load measurement and interpretation | |
| Alex | Hall | | DHSc | Department of Emergency Medicine, Emory University School of Medicine | Atlanta, GA, USA | Site Investigator | |
| David | Wright | | MD | Department of Emergency Medicine, Emory University School of Medicine | Atlanta, GA, USA | Site PI | |
| Ronald A | Reilkoff | | MD | Division of Pulmonary, Allergy, Critical Care and Sleep Medicine, Department of Medicine, University of Minnesota | Minneapolis, MN, USA | Site Investigator | |
| Tyler | Bold | | MD PhD | Division of Infectious Diseases, Department of Medicine, University of Minnesota | Minneapolis, MN, USA | Oversaw biospecimen collection, processing, and analysis | |
| Kenneth | Beckman | | PhD | University of Minnesota Genomics Center | Minneapolis, MN, USA | Oversaw Viral load measurement and interpretation | |
| Ryan | Langlois | | PhD | Department of Microbiology and Immunology, University of Minnesota | Minneapolis, MN, USA | Assisted with biospecimen collection, processing, and analysis | |

Supplemental Online Content: Nonauthor Collaborators

*Indicates required information. Only first name, last name, and suffix will appear in PubMed.

| ***First Name and Middle Initial(s)** | ***Last Name** | ***Suffix (eg, Jr, III)** | Academic Degrees | Institution | Location (city, state/province, country) | Role or Contribution, eg, chair, principal investigator | Group (if more than 1 Group listed in the byline) and/or Subgroup (eg, Steering Committee) |
|---|---|---|---|---|---|---|---|
| Matthew T | Aliota | | PhD | Department of Veterinary and Biomedical Sciences, University of Minnesota | Twin Cities, St. Paul, MN, USA | Assisted with biospecimen collection, processing, and analysis | |
| James | Galbriath | | MD | Department of Emergency Medicine, University of Mississippi Medical Center | Jackson, MS USA | Site Investigator | |
| Margaret | Beyer | | BS | Department of Emergency Medicine, Henry Ford Hospital, Wayne State University | Detoit, MI, USA | Study Coordinator | |
| Chas | Salmen | | MD | Department of Surgery, University of Minnesota | Minneapolis, MN, USA | Site Investigator | |
| Dana | Byrne | | MD | Department of Internal Medicine, Cooper University Health Care | Camden, NJ, USA | Site PI | |
| Brian | Roberts | | MD | Department of Emergency Medicine, Cooper University Health Care | Camden, NJ, USA | Site Investigator | |
