# Supplementary material for: Efficacy of Losartan in Hospitalized Patients With COVID-19–Induced Lung Injury: A Randomized Clinical Trial
Source: JAMA Netw Open. 2022 Mar 16;5(3):e222735. doi: 10.1001/jamanetworkopen.2022.2735 (PMC8928006; doi:10.1001/jamanetworkopen.2022.2735)
Supplement: Supplement 4. — Data Sharing Statement [file jamanetwopen-e222735-s004.pdf]

Data Sharing Statement

Puskarich. Efficacy of Losartan in Hospitalized Patients With COVID-19-Induced Lung Injury. *JAMA Netw Open*. Published March 16, 2022. doi:10.1001/jamanetworkopen.2022.2735

Data
Data available: Yes
Data types: Deidentified participant data
How to access data: University of Minnesota Data Repository (DRUM)
When available: beginning date: 07-01-2022

Supporting Documents
Document types: None

Additional Information
Who can access the data: N/A
Types of analyses: N/A
Mechanisms of data availability: N/A
Any additional restrictions: N/A
